# Genome-wide association study considering genotype-by-environment interaction for productive and reproductive traits using whole-genome sequencing in Nellore cattle

**DOI:** 10.1186/s12864-024-10520-x

**Published:** 2024-06-20

**Authors:** Ivan Carvalho Filho, Leonardo M. Arikawa, Lucio F. M. Mota, Gabriel S. Campos, Larissa F. S. Fonseca, Gerardo A. Fernandes Júnior, Flavio S. Schenkel, Daniela Lourenco, Delvan A. Silva, Caio S. Teixeira, Thales L. Silva, Lucia G. Albuquerque, Roberto Carvalheiro

**Affiliations:** 1 Department of Animal Science, School of Agricultural and Veterinarian Sciences, São Paulo State University (UNESP), Jaboticabal, SP 14884-900 Brazil; 2 Centre for Genetic Improvement of Livestock, Department of Animal Biosciences, University of Guelph, Guelph, Ontario, N1G2W1 Canada; 3 Department of Animal and Dairy Science, University of Georgia, Athens, GA 30602 USA; 4 National Council for Science and Technological Development, Brasilia, DF 71605-001 Brazil

## Abstract

**Background:**

The genotype-by-environment interaction (GxE) in beef cattle can be investigated using reaction norm models to assess environmental sensitivity and, combined with genome-wide association studies (GWAS), to map genomic regions related to animal adaptation. Including genetic markers from whole-genome sequencing in reaction norm (RN) models allows us to identify high-resolution candidate genes across environmental gradients through GWAS. Hence, we performed a GWAS via the RN approach using whole-genome sequencing data, focusing on mapping candidate genes associated with the expression of reproductive and growth traits in Nellore cattle. For this purpose, we used phenotypic data for age at first calving (AFC), scrotal circumference (SC), post-weaning weight gain (PWG), and yearling weight (YW). A total of 20,000 males and 7,159 females genotyped with 770k were imputed to the whole sequence (29 M). After quality control and linkage disequilibrium (LD) pruning, there remained ∼ 2.41 M SNPs for SC, PWG, and YW and ∼ 5.06 M SNPs for AFC.

**Results:**

Significant SNPs were identified on *Bos taurus* autosomes (BTA) 10, 11, 14, 18, 19, 20, 21, 24, 25 and 27 for AFC and on BTA 4, 5 and 8 for SC. For growth traits, significant SNP markers were identified on BTA 3, 5 and 20 for YW and PWG. A total of 56 positional candidate genes were identified for AFC, 9 for SC, 3 for PWG, and 24 for YW. The significant SNPs detected for the reaction norm coefficients in Nellore cattle were found to be associated with growth, adaptative, and reproductive traits. These candidate genes are involved in biological mechanisms related to lipid metabolism, immune response, mitogen-activated protein kinase (MAPK) signaling pathway, and energy and phosphate metabolism.

**Conclusions:**

GWAS results highlighted differences in the physiological processes linked to lipid metabolism, immune response, MAPK signaling pathway, and energy and phosphate metabolism, providing insights into how different environmental conditions interact with specific genes affecting animal adaptation, productivity, and reproductive performance. The shared genomic regions between the intercept and slope are directly implicated in the regulation of growth and reproductive traits in Nellore cattle raised under different environmental conditions.

**Supplementary Information:**

The online version contains supplementary material available at 10.1186/s12864-024-10520-x.

## Background

Nellore cattle are raised under different production systems that are predominantly characterized by extensive pastures, with animals being influenced by a wide range of climatic conditions. These environmental variations introduce disparities in forage availability and quality, as well as challenges related to heat stress, among other factors. In this context, differences between the production systems of the selected herds and commercial herds can result in differences in productive performance, which has significant economic implications for the livestock industry [1, 2]. These heterogeneous environmental conditions can decrease the accuracy of breeding values when genotype-by-environment (GxE) interactions are not accounted for during genetic evaluation [3, 4].

GxE interactions may affect the reranking of animals across different environments [5] and have been identified as an important source of variation in the productive and reproductive performance of beef cattle [3, 4, 6, 7]. The evaluation of GxE interactions in beef cattle is routinely performed using reaction norm models [8, 9] to predict breeding values under different environmental conditions and to assess environmental sensitivity [1]. Traditionally, environmental gradients (EG) used to evaluate GxE interactions have been derived from contemporary group (CG) solutions based on phenotypic information [3, 7, 10]. This is because the CG encompasses the differences in nutritional and climatic factors, as well as the management in which the animals were raised over a determined period, representing a key factor in phenotypic variability [11]. Integrating genetic markers into reaction norm models allows the identification of candidate genes along environmental gradients through genome-wide association studies (GWAS) [12]. Moreover, the advent of whole-genome sequencing (WGS) technology has made it possible to refine the identification of genomic regions that affect the traits of interest by providing greater chances of identifying causal mutations when compared to marker panels with medium or high density [13]. Therefore, the combination of GWAS with WGS enables the unraveling of important regions of the genome, as well as candidate genes, thereby enabling the development of more informative marker panels and conducting more accurate genomic evaluations [13].

Implementing the reaction norm model with GWAS analysis could lead to a greater understanding of the genetic and physiological mechanisms regulating economically important traits. This approach also facilitates the identification of candidate genes associated with these traits across diverse environmental conditions. Thus, the overarching aim of this study was to perform GWAS utilizing sequencing data, focusing on mapping candidate genes associated with the expression of reproductive and growth traits in Nellore cattle, employing the reaction norm approach.

## Materials and methods

### Phenotypic data

Phenotypic information was obtained from 138,706 females and 942,577 Nellore males born between 1984 and 2019 and belonging to three commercial breeding programs (DeltaGen, Cia do Melhoramento, and Paint – CRV Lagoa) in Brazil and Paraguay that integrate the Nellore Alliance dataset (www.gensys.com.br*).*

The traits used in the present study were age at first calving (AFC), scrotal circumference (SC), post-weaning weight gain (PWG), and yearling weight (YW). In reproductive management, some herds exposed heifer to reproduction in two breeding seasons: (1) heifers aged 16 months are exposed to reproduction for 60 days in an anticipated breeding season from February to April to identify sexually precocious heifers and (2) heifers that were not pregnant during the anticipated breeding season were given another opportunity during the regular breeding season (October and January), usually at approximately 24 months. During the mating season, the heifers were either artificially inseminated or naturally mated (∼ 50%). When a fixed-time AI protocol was used, the entire contemporary group received the same protocol, and pregnancy was diagnosed approximately 60 days after the end of the breeding season. Non-conceiving females are discarded from the herd. The AFC was computed in days, which is the difference between the first calving date and the heifers’ birth date. SC was measured in centimeters (cm) at yearling, and PWG was calculated in kilograms (kg) to determine the difference between the YW and weaning weight.

For the analysis, only animals with known sires and dams and from contemporary groups (CG) with a minimum of 20 animals were considered. The CG for the evaluated traits considered animals from the same year and season of birth, herd (at birth, weaning, and yearling), and management group (at birth, weaning, and yearling). The management group includes information about nutritional and sanitary treatment at each growth stage. For YW and PWG, sex was also added to the CG. Descriptive statistics of the dataset used for each trait after data editing are shown in Table 1.

**Table 1:** Descriptive statistics of phenotypic information for age at first calving (AFC), scrotal circumference (SC), post-weaning gain (PWG), and yearling weight (YW)

Traits	*N*	*N* Female	*N* Male	Mean	Min	Max	SD	CG
AFC (days)	138,706	138,706	-	1,012	544	1,220	132	3,861
SC (cm)	438,592	-	438,592	26.7	15	45	3.8	9,988
PWG (kg)	920,981	470,732	450,249	100.8	30	250	36	20,926
YW (kg)	942,577	483,691	458,886	287.9	200	542	48.5	21,317

N: number of observations; Min and Max: minimum and maximum values; SD: standard deviation and GC: number of contemporary groups

The pedigree dataset considered genealogical information of 1,578,503 individuals form 9946 sires and 628,231 dams encompassing 95,606 populations. The pedigree data set had an average inbreeding of 0.16% in the whole population, and the proportion of inbreeding animals was 2.66% (42,026 animals) over the total inbreeding population, showing an inbreeding average of 2.56% (0.01 – 27.10%).

### Genomic data

A total of 51,485 animals were genotyped with the Illumina BovineHD (HD) chip (∼ 778 K SNPs; 4,559 samples) or with a lower and medium density assay (from ∼ 26 K to ∼ 74 K SNPs; 46,926 samples). Animals genotyped at lower and medium densities were imputed to HD panels using the software FImpute v.3 [14] considering the ARS-UCD1.2 map. Additionally, 151 influential Nellore sires were whole genome sequenced (WGS) using the Illumina HiSeq X™ Ten (*n* = 51) and Illumina NovaSeq 6000 (*n* = 100) platforms at an average sequence coverage of 14.5x (from 7.8 to 26.3x). Quality control, alignment, and variant calling were carried out following the guidelines provided by the 1000 Bull Genomes Project and described by Fernandes Júnior et al. [15]. A total of 30,394,484 autosomal SNP markers remained after quality control. Animals genotyped with 700k were imputed for WGS using the software FImpute v.3 [14], considering as a reference population 151 sires with the highest number of genotyped animals. The imputation accuracy of 0.94 was previously evaluated; for more details see Fernandes Júnior et al. [15].

Due to computational limitations, we selected 20,000 genotypes for SC, PWG, and YW and 7,159 genotypes for AFC with GEBV accuracy higher than 0.70. The GEBVs accuracy was calculated based on prediction error variance (PEV) and the genetic variance for each trait (${{\sigma }}_{\text{a}}^{2}$) using the following equation [16]: $\text{A}\text{c}\text{c}=1-\sqrt{\text{P}\text{E}\text{V}/{{\sigma }}_{\text{a}}^{2}}$. The GEBV was estimated using the following animal model:

$$\mathbf{y}=\mathbf{X}\mathbf{b}+ \mathbf{Z}\mathbf{a}+\mathbf{e}$$,

where $\mathbf{y}$ is the vector of observations;$\mathbf{b}$ is the vector of fixed effects of CG and age of the animal at the measurement as linear and quadratic covariates for YW and PWG; $\mathbf{a}$ is the vector of genetic additive effects, and $\mathbf{e}$ is the vector of random residual effects. The $\mathbf{X}$ and $\mathbf{Z}$ are the incidence matrices related to fixed (**b**) and random effects (**a**), respectively. The model was fitted considering the random effects of animals and residuals as normally distributed: $\mathbf{a}\sim\text{N}(0,\mathbf{A}{{\sigma }}_{\text{a}}^{2}$ and $\mathbf{e}\sim\text{N}(0, \mathbf{I}{{\sigma }}_{\text{e}}^{2}$), where **A** is the numerator relationship matrix between animals, **I** is the identity matrix;${{\sigma }}_{\text{a}}^{2}$ is the additive genetic variance and ${{\sigma }}_{\text{e}}^{2}$ is the residual variance. The parameters were estimated using the restricted maximum likelihood method considering the average information algorithm implemented in blupf90+ software [17].

Considering the number of animals genotyped for each trait and a large number of markers (30,394,484), markers with linkage disequilibrium values (r^2^) greater than 0.75 for SC, PWG, and YW and greater than 0.95 for AFC were pruned using PLINK 2.0 [18]. This strategy was used to adjust the number of genotyped animals and genetic markers to the computational capacity. Additionally, quality control (QC) of the genomic information was performed by removing autosomal markers with a minor allele frequency (MAF) lower than 0.05, Hardy–Weinberg equilibrium (*P* ≤ 10^− 5^), and a call rate of markers and samples lower than 0.90. After quality control and removing markers for LD, a total of ∼ 2.41 M SNPs for SC, PWG, and YW and ∼ 5.06 M SNPs for AFC remained for the GWAS analyses via reaction norm models.

### Genotype by environment interaction (GxE)

#### Environmental gradient descriptor

The dataset used to evaluate the sensitivity of sexual precocity indicators (AFC and SC) and growth traits (YW and PWG) was assessed through the reaction norm model in two steps [3, 4]. In the first step, the environmental gradients (EG) for AFC, SC, and YW were based on the best linear unbiased estimates (BLUE) solutions of CG for YW. We focused on YW because differences in production environments affecting YBW have a significant impact on heifers’ early sexual maturity [3, 12, 19]. The EG for PWG was based on its CG solutions. The EG was obtained with an animal model as follows:

$$\mathbf{y}=\mathbf{X}\mathbf{b}+ \mathbf{Z}\mathbf{a}+\mathbf{e}$$

where $\mathbf{y}$ is the vector of observations for YW or PWG;$\mathbf{b}$ is the vector of fixed effects of CG and age of the animal at the measurement as linear and quadratic covariates; $\mathbf{a}$ is the vector of genetic additive effects assumed to follow a normal distribution given by $\text{N}(0,\mathbf{A}{{\sigma }}_{a}^{2}$) and $\mathbf{e}$ is the vector of random residual effects considered normally distributed as $\text{N}(0,\mathbf{I}{{\sigma }}_{\text{e}}^{2}$),. The $\mathbf{X}$ and $\mathbf{Z}$ are the incidence matrices related to fixed (**b**) and random effects (**a**), respectively. The model was performed using the blupf90 + software [17].

The EG descriptors obtained by CG solutions were standardized to a mean value of 0 and standard deviation (SD) equal to 1, with values ranging from − 3 to + 3 SD, to keep the environmental gradients on the same scale. The CG solutions of YW for AFC ranged from 228.98 (low EG; -3 SD) to 342.09 (high EG, 3 SD). The CG solutions of YW for young bulls with SC information varied from 244.17 (low EG; -3 SD) to 388.23 (high EG, 3 SD), and for animals with YW varied from 227.46 (low EG; -3 SD) to 390.22 (high EG, 3 SD). The CG solutions of PWG for PWG ranged from 55.55 (low EG; -3 SD) to 177.43 (high EG, 3 SD).

### Reaction norm (RN) model

In the second step, a single-step genomic reaction norm (ssGRN) model was used to assess GxE. The model assumed a heterogeneous residual variance across EG, using linear regression on ${EG}_{i}$, with the intercept and slope coefficients being modeled using the log-residual function [20].

$${{\mathbf{y}}_{\mathbf{i}\mathbf{j}}= \mathbf{X}\mathbf{b}+\phi{\mathbf{E}\mathbf{G}}_{\mathbf{i}}+{\varvec{Z}}_{0}{{\mathbf{a}}_{0}}_{\mathbf{j}}+ {\varvec{Z}}_{1}{{\mathbf{a}}_{1}}_{\mathbf{j}}+{\mathbf{e}}_{\mathbf{i}\mathbf{j}}}$$

where: ${\mathbf{y}}_{\mathbf{i}\mathbf{j}}$ is the phenotypic information (AFC, SC, YW, and PWG) of animal j on the environment i; $\mathbf{b}$ is the vector of fixed effects of CG for all traits and age at measurement as linear and quadratic covariates for SC, YW, and PWG; $\phi$ is the fixed regression coefficient of ${\mathbf{y}}_{\mathbf{i}\mathbf{j}}$ on ${\text{E}\text{G}}_{\text{i}}$; ${{\mathbf{a}}_{0}}_{\mathbf{j}}$ is the additive genetic effect for the intercept of animal j, ${{\mathbf{a}}_{1}}_{\mathbf{j}}$ is the additive effect of the slope of the animal j and ${\mathbf{e}}_{\mathbf{i}\mathbf{j}}$is residual effects. The **X**, ${\varvec{Z}}_{0}$ and ${\varvec{Z}}_{1}$ are the incidence matrix relating the fixed effects (**b**), intercept (${\mathbf{a}}_{0}$) and slope (${\mathbf{a}}_{1}$**)** to **y**. The ssGRNM model was fitted considering the following assumptions:

$$\left[ {\begin{array}{*{20}{c}}{{{\text{a}}_0}} \\ {{{\text{a}}_1}} \end{array}} \right] \sim {\text{N}}\left( {0,{\mathbf{H}} \otimes \left[ {\begin{array}{*{20}{c}}{\sigma _{{{\text{a}}_0}}^2}&{{\sigma _{{{\text{a}}_0}{{\text{a}}_1}}}} \\ {{\sigma _{{{\text{a}}_0}{{\text{a}}_1}}}}&{\sigma _{{{\text{a}}_1}}^2} \end{array}} \right]} \right)\,{\text{and}}\,{{\text{e}}_{{\text{ij}}}} \sim {\text{N}}(0,{\mathbf{I}} \otimes {\mathbf{R}})$$

where **H** is a combined pedigree-genomic relationship matrix, ${\sigma }_{{a}_{0}}^{2}$ and ${\sigma }_{{a}_{1}}^{2}$ are the genetic variances for intercept and slope, respectively, ${\sigma }_{{a}_{0}{a}_{1}}$ is the genetic covariance between the reaction norm parameters (intercept and slope), ⊗ is the Kronecker product; **I** is an identity matrix, and $\mathbf{R}$ is the residual variance matrix considering heterogeneous classes. In the ssGRN methodology, the inverse of the **H** matrix (${\mathbf{H}}^{-1}$) is given as follows:

$${\varvec{H}}^{-1}= {\varvec{A}}^{-1}+ \left[\begin{array}{cc}0 & 0\\ 0& {\varvec{G}}^{-1}- {\varvec{A}}_{22}^{-1}\end{array} \right]$$

where **A**^− 1^ is the inverse of the pedigree-based relationship matrix for all animals, ${\varvec{A}}_{22}^{-1}$ is the inverse of the pedigree-based relationship matrix for the genotyped animals, and **G**^− 1^ is the inverse of the genomic relationship matrix (**G**), obtained according to VanRaden [21]:

$$G = \frac{{WW'}}{{\sum _{i = 1}^w2{p_i}(1 - {p_i})}}$$

where $\mathbf{W}$ is the genotype matrix with codes 0, 1, and 2 for AA, AB, and BB, adjusted for allele frequency expressed as $2{\text{p}}_{\text{i}}$, and ${\text{p}}_{\text{i}}$ is the frequency of the second allele. These analyses were performed using the software blupf90 + from the BLUPF90 [17].

The p-values associated with the SNP effects were obtained from the postGSf90 program within the BLUPF90 software suite [17]. The p-values for the SNP effects were obtained by Aguilar et al. [22]:

$$\text{p}-\text{v}\text{a}\text{l}\text{u}\text{e}=2 \left(1- {\upvarphi }\left(\frac{\left|{{\upalpha }}_{\text{i}}\right|}{\text{S}\text{D}\left({{\upalpha }}_{\text{i}}\right)}\right)\right)$$

where ${{\upalpha }}_{\text{i}}$ is the allele substitution effect of the ith marker, $\text{S}\text{D}\left({{\upalpha }}_{\text{i}}\right)$ is the standard deviation of the ith SNP marker (${{\upalpha }}_{\text{i}}$) and ${\upvarphi }$ is cumulative function of the normal distribution.

### Multiple testing correction and significance testing

The Bonferroni correction test was performed considering a significance threshold for the marker of 0.05 divided by the number of independent BTA segments (Me). The Me considered the effective population size (Ne) and the BTA length [L, in centimorgans (cM)] and was calculated as proposed by Goddard et al. [23]: $\text{M}\text{e}= 2\text{N}\text{e}\text{L}/(\text{l}\text{o}\text{g}(\text{N}\text{e}\text{L}\left)\right)$, where Ne was equal to 100 [24], and L equal to 2,750 cM for the autosomal chromosome of Nellore cattle (https://ncbi.nlm.nih.gov/datasets/genome/GCF_000247795.1/). As a result, SNP were deemed statistically significant if their${-\text{l}\text{o}\text{g}}_{10}\left(\text{p}-\text{v}\text{a}\text{l}\text{u}\text{e}\right)$ was greater than 5.45. The inflation/deflation factor (${\uplambda }$) were calculated as ${\uplambda }=\text{m}\text{e}\text{d}\text{i}\text{a}\text{n}\left({-\text{l}\text{o}\text{g}}_{10}\left(\text{p}-\text{v}\text{a}\text{l}\text{u}\text{e}\right)\right)/0.456$, and λ values varied from 0.95 to 1.18 were considered acceptable in GWAS [25].

### Functional analysis

After GWAS analyses, all SNPs were ranked based on their p-values. The average distance in bases pair between SNPs in each BTA was closer to 1 kb (see additional File 1 Table S1). Due to the short distance between genetic markers, a region of ± 5 kb around each significant SNP marker was used to map the genes using the Ensembl Variant Effect Predictor (VEP) [26] considering the ARS-UCD1.2 assembly as the reference genome (GCA_002263795.2).

A “training list” containing the top 100 genes associated with relevant keywords for each trait (see Additional file 1 Table S2) and for GxE (resilience, resistance, robustness, fitness, plasticity, and adaptability) was created using Guildify [27]. The gene list from VEP and training list from Guildify were used as a test list in the ToppGene Suite [28]. The prioritized significant genes were selected based on a multiple correction false discovery rate (FDR) of 5% (p-value ≤ 10 − 3), indicating that the test genes have the same functional profile as the genes on the “trained” list [28]. The R packages ClusterProfiler [29] and enrichplot [30] were used for enrichment analysis and functional clustering of GO terms for the list of “test” genes. Genes and GO terms were considered enriched when the FDR was lower than 5%.

## Results

### Significant markers

**Fig. 1:**
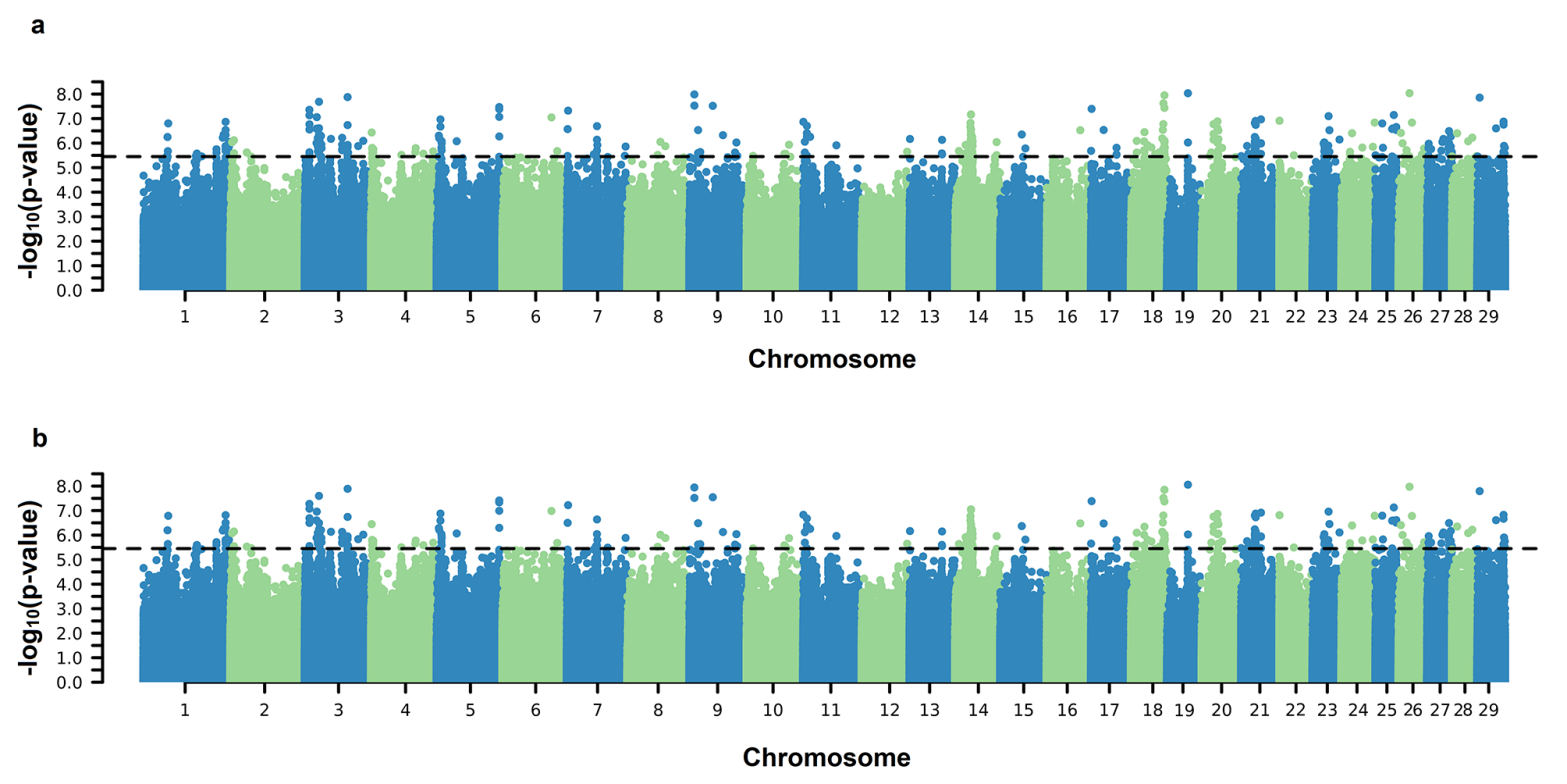
Manhattan plots of ${-\text{l}\text{o}\text{g}}_{10}(\text{p}-\text{v}\text{a}\text{l}\text{u}\text{e})$ for the intercept (**a**) and slope (**b**) coefficients of the reaction norm model for age at first calving (AFC). The horizontal line represents the significance threshold ${-\text{l}\text{o}\text{g}}_{10}\left(\text{p}-\text{v}\text{a}\text{l}\text{u}\text{e}\text{d}\right)>5.45$ used to identify the significant SNPs

**Fig. 2:**
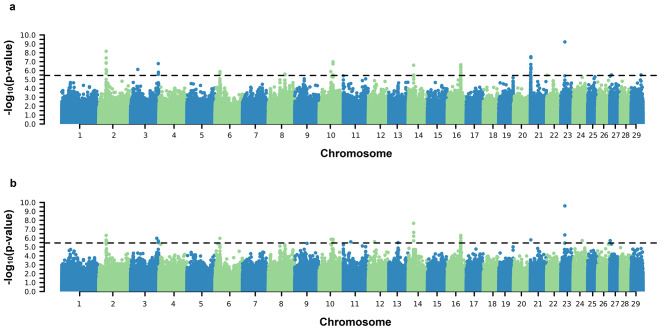
Manhattan plots of ${-\text{l}\text{o}\text{g}}_{10}(\text{p}-\text{v}\text{a}\text{l}\text{u}\text{e})$ for the intercept (**a**) and slope (**b**) coefficients of the reaction norm model for scrotal circumference (SC). The horizontal line represents the significance threshold ${-\text{l}\text{o}\text{g}}_{10}\left(\text{p}-\text{v}\text{a}\text{l}\text{u}\text{e}\text{d}\right)>5.45$ used to identify the significant SNPs

**Fig. 3:**
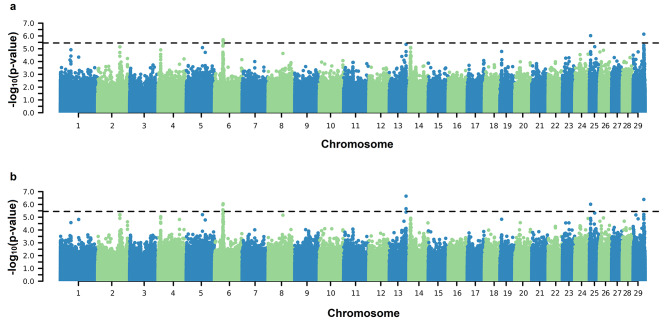
Manhattan plots of ${-\text{l}\text{o}\text{g}}_{10}(\text{p}-\text{v}\text{a}\text{l}\text{u}\text{e})$ for the intercept (**a**) and slope (**b**) coefficients of the reaction norm model for post-weaning weight gain (PWG). The horizontal line represents the significance threshold ${-\text{l}\text{o}\text{g}}_{10}\left(\text{p}-\text{v}\text{a}\text{l}\text{u}\text{e}\text{d}\right)>5.45$ used to identify the significant SNPs

**Fig. 4:**
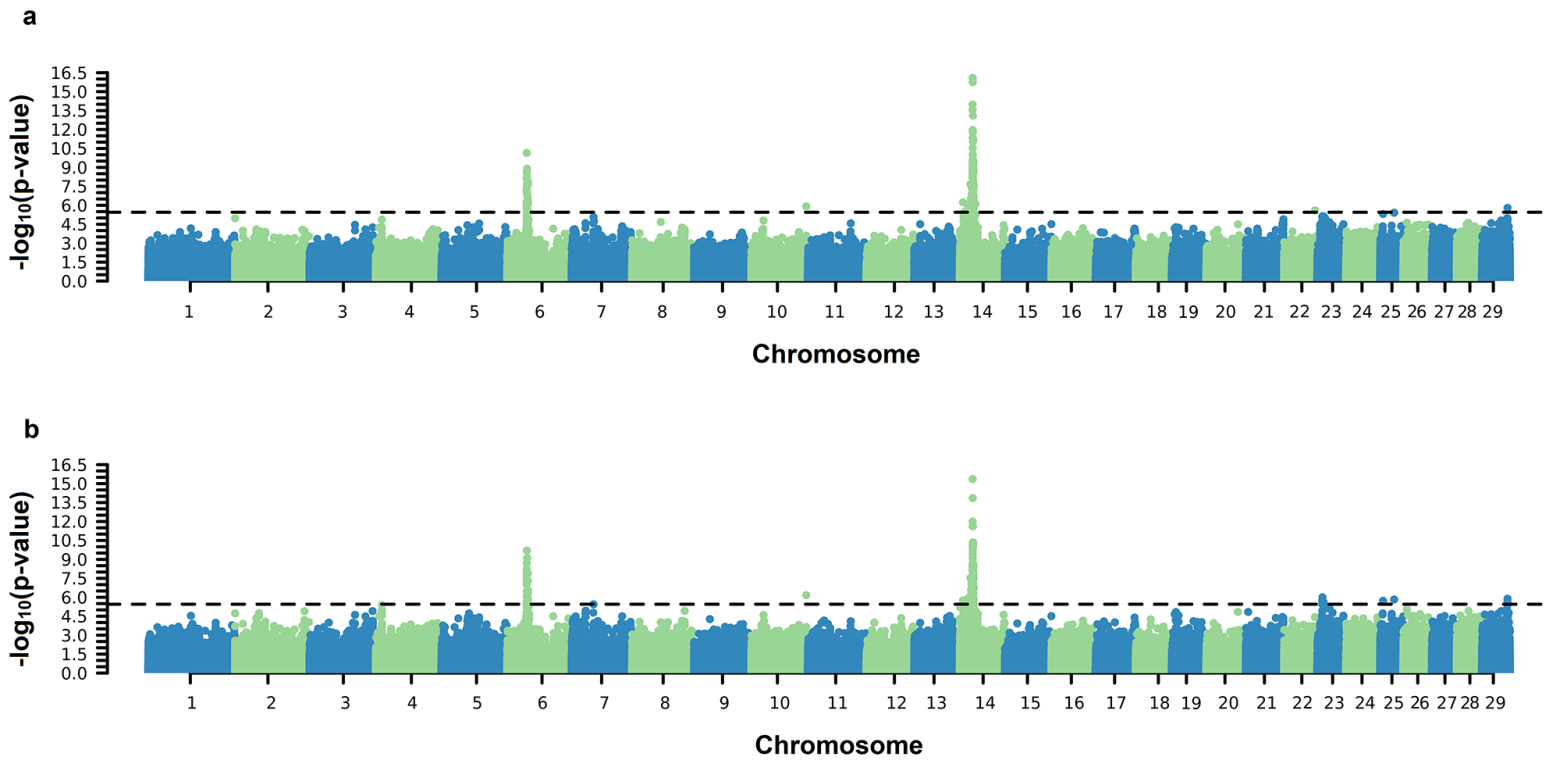
Manhattan plots of ${-\text{l}\text{o}\text{g}}_{10}(\text{p}-\text{v}\text{a}\text{l}\text{u}\text{e})$ for the intercept (**a**) and slope (**b**) coefficients of the reaction norm model for yearling weight (YW). The horizontal line represents the significance threshold ${-\text{l}\text{o}\text{g}}_{10}\left(\text{p}-\text{v}\text{a}\text{l}\text{u}\text{e}\text{d}\right)>5.45$ used to identify the significant SNPs

Significant SNPs associated with both the AFC intercept and slope on EG coefficients were identified on practically all BTAs except for BTA12 (Fig. 1). Significant SNPs were found on BTAs 2, 3, 6, 10, 14, 16, 21, and 23 for both SC coefficients (Fig. 2). For PWG, significant SNPs were identified on BTA 6, 25, and 29 for intercept and on BTA 6, 13, 25, and 29 for the slope coefficient (Fig. 3). For YW, significant markers were found on BTA 6, 10, 14, and 29 for the intercept coefficient and on BTA 6, 10, 14, 23, 25, and 29 for the slope (Fig. 4). Considering a region of ± 5 kb of the significant SNPs, a total of 56, 9, and 24 positional candidate genes were identified for intercept coefficient affecting AFC (see Additional file 1 Table S3), SC (see Additional File 1 Table S4) and YW (see Additional file 1 Table S6), respectively, while for PWG (see Additional file 1 Table S5) no gene was found for the intercept. For the slope coefficient, a total of 50, 10, 3, and 29 genes were identified as affecting the AFC, SC, PWG, and YW, respectively (see Additional File 1 Table S3–S6).

**Fig. 5:**
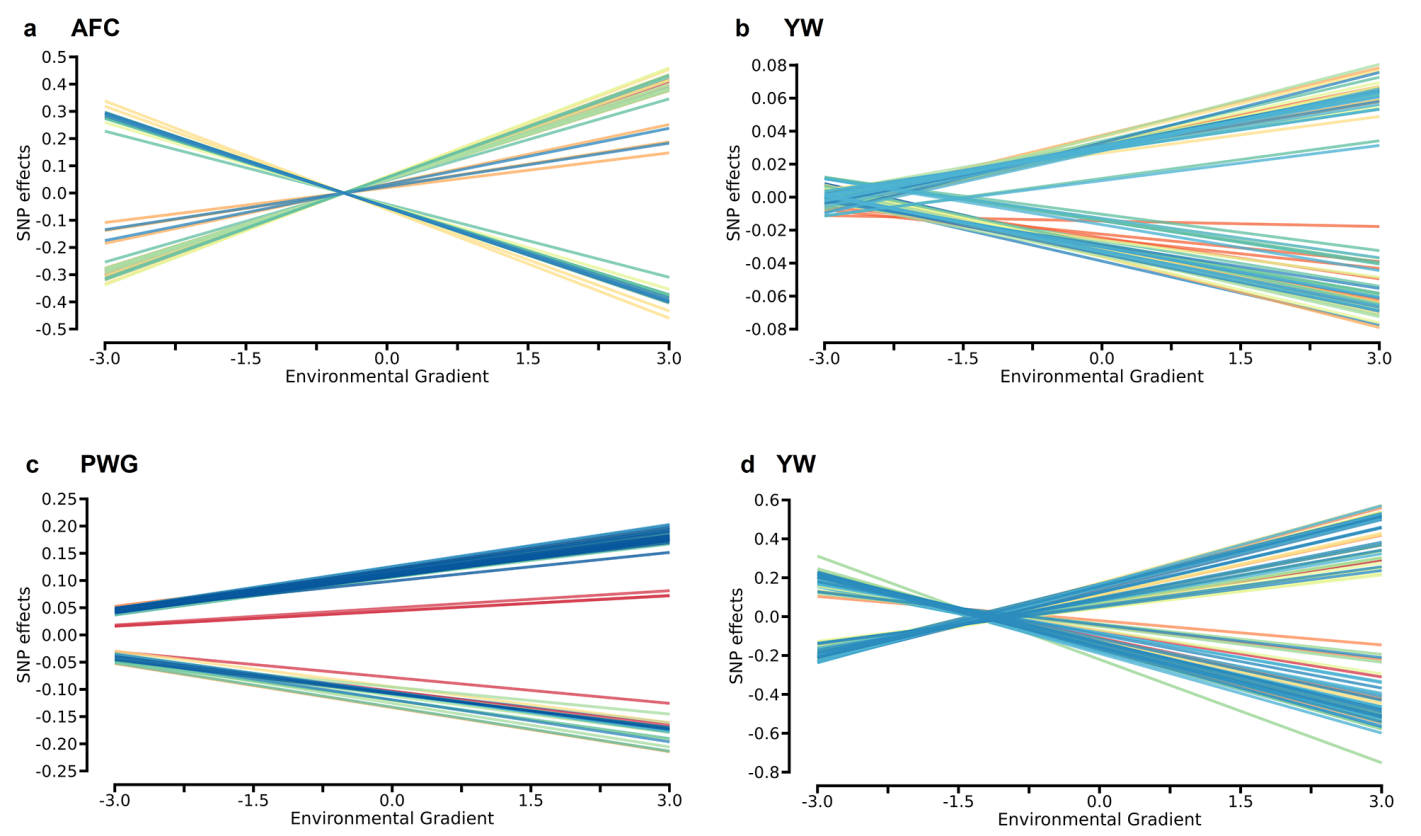
Single nucleotide polymorphism (SNP) effect estimates significantly associated (${-\text{l}\text{o}\text{g}}_{10}\left(\text{p}-\text{v}\text{a}\text{l}\text{u}\text{e}\text{d}\right)>5.45$) with age at first calving (AFC, **a**), scrotal circumference (SC, **b**), post-weaning weight gain (PWG, **c**) and for yearling weight (YW, **d**) across environmental conditions. Different colors represent the chromosome where the SNP marker was identified

The significant SNP markers (− log10 (p-value) > 5.45) for productive and reproductive traits in Nellore cattle were environmentally dependent, with reranking of their effects across EG levels (Fig. 5). The SNP makers effects in the low EG (-3.0) were different from those in the high EG (3.0, Fig. 5). This strong effect of SNPxE interaction indicates that genomic regions have a striking effect on the Nellore sexual precocity indicator (Fig. 5a and b) and weight traits (Fig. 5c and d) at a determined EG level, with changes not only in magnitude but also in direction. A greater dispersion of SNP marker effects was observed for SC (Fig. 5b) and YW (Fig. 5d) when the EG level became less restrictive.

After gene prioritization by ToppGene, 32, 6, and 2 positional candidate genes were retained for AFC, SC, and YW intercept coefficient, respectively. For the slope, there were 31, 6, 1, and 3 genes for AFC (Table 2), SC, PWG, and YW (Table 3), respectively. In the functional analysis, enriched clusters representing the relationships between prioritized genes and GO terms for intercept and slope common genes were found for the studied traits, and the complete table with all enrichment analysis results can be found in the supplementary material (Tables S7 to S9).

**Table 2:** Prioritized candidate gene list for age at first calving (AFC) identified by Guildify and ToppGene analysis

Coefficient	SNP	BTA^1^	Position^2^	MAF^3^	*P*-value^4^	Gene	Adjusted *P*-value^5^
Intercept	rs43230433	1	44,063,185	0.035	0.00000113	*COL8A1*	0.016
	rs381434568	1	150,106,131	0.516	0.00000265	*KCNJ6*	< 0.0000001
	rs110470442	3	8,634,391	0.756	0.00000056	*ITLN2*	0.009
	rs379055283	5	9,003,285	0.653	0.00000167	*SYT1*	< 0.0000001
	rs720484023	5	9,385,330	0.278	0.00000167	*PPP1R12A*	0.005
	rs715728654	7	55,339,011	0.424	0.00000145	*KCTD16*	0.048
	rs517426012	8	69,530,246	0.335	0.00000267	*BMP1*	0.012
	rs445538954	10	77,658,449	0.537	0.00000233	*FUT8*	0.014
	rs519493432	11	12,923,464	0.034	0.00000111	*DYSF*	0.014
	rs136849909	13	1,263,836	0.878	0.00000136	*PLCB1*	0.003
	rs523777757	13	59,836,380	0.334	0.00000147	*SNPH*	0.011
	rs208954324	14	19,491,973	0.825	0.00000238	*PRKDC*	0.009
	rs520454646	14	29,257,049	0.388	0.00000085	*CYP7B1*	0.014
	rs723811441	14	31,027,180	0.777	0.00000256	*SGK3*	0.009
	rs43195263	14	32,141,429	0.863	0.00000259	*PREX2*	0.019
	rs133920327	14	76,084,780	0.177	0.00000182	*CNGB3*	0.014
	rs521155455	18	10,690,957	0.243	0.00000163	*MEAK7*	0.026
	rs134265088	19	38,330,461	0.856	0.00000002	*SKAP1*	0.011
	rs456261266	20	30,542,035	0.050	0.00000138	*FGF10*	< 0.0000001
	rs720222086	20	37,304,855	0.823	0.00000313	*NIPBL*	0.011
	rs380578567	21	25,211,390	0.046	0.00000030	*CTSH*	0.002
	rs210224020	21	26,556,144	0.821	0.00000025	*ABHD17C*	0.014
	rs475843527	21	26,731,693	0.546	0.00000025	*CEMIP*	0.014
	rs519423782	24	57,664,815	0.063	0.00000283	*ALPK2*	0.014
	rs464263309	25	33,683,982	0.366	0.00000014	*BAZ1B*	0.014
	rs42102555	26	46,691,693	0.132	0.00000332	*DOCK1*	0.009
	rs714730097	27	7,469,393	0.170	0.00000350	*GPM6A*	0.005
	rs453588982	27	40,147,406	0.836	0.00000197	*TOP2B*	0.048
	rs382211336	27	40,216,810	0.147	0.00000337	*RARB*	0.003
	rs715500178	28	29,695,289	0.474	0.00000168	*NDST2*	0.048
	rs720113004	29	34,597,655	0.039	0.00000050	*NTM*	0.048
	rs520498826	29	49,201,864	0.894	0.00000259	*CD81*	0.009
Slope	rs43230433	1	44,063,185	0.035	0.00000127	*COL8A1*	0.016
	rs381434568	1	150,106,131	0.516	0.00000314	*KCNJ6*	< 0.0000001
	rs110470442	3	8,634,391	0.756	0.00000064	*ITLN2*	0.009
	rs379055283	5	9,003,285	0.653	0.00000181	*SYT1*	< 0.0000001
	rs720484023	5	9,385,330	0.278	0.00000219	*PPP1R12A*	0.005
	rs715728654	7	55,339,011	0.424	0.00000182	*KCTD16*	0.044
	rs517426012	8	69,530,246	0.335	0.00000262	*BMP1*	0.013
	rs445538954	10	77,658,449	0.537	0.00000266	*FUT8*	0.014
	rs519493432	11	12,923,464	0.034	0.00000112	*DYSF*	0.015
	rs136849909	13	1,263,836	0.978	0.00000137	*PLCB1*	0.003
	rs523777757	13	59,836,380	0.334	0.00000142	*SNPH*	0.011
	rs208954324	14	19,491,973	0.825	0.00000244	*PRKDC*	0.009
	rs520454646	14	29,257,049	0.388	0.00000109	*CYP7B1*	0.014
	rs723811441	14	31,027,180	0.777	0.00000264	*SGK3*	0.009
	rs43195263	14	32,141,429	0.863	0.00000267	*PREX2*	0.018
	rs133920327	14	76,084,780	0.177	0.00000219	*CNGB3*	0.014
	rs521155455	18	10,690,957	0.243	0.00000160	*MEAK7*	0.026
	rs134265088	19	38,330,461	0.856	0.00000002	*SKAP1*	0.011
	rs456261266	20	30,542,035	0.050	0.00000175	*FGF10*	< 0.0000001
	rs380578567	21	25,211,390	0.046	0.00000032	*CTSH*	0.002
	rs210224020	21	26,556,144	0.821	0.00000028	*ABHD17C*	0.014
	rs475843527	21	26,731,693	0.546	0.00000027	*CEMIP*	0.014
	rs110296566	24	19,710,489	0.026	0.00000081	*CELF4*	0.048
	rs519423782	24	57,664,815	0.063	0.00000305	*ALPK2*	0.014
	rs464263309	25	33,683,982	0.366	0.00000015	*BAZ1B*	0.014
	rs453588982	27	40,147,406	0.836	0.00000202	*TOP2B*	0.044
	rs382211336	27	40,216,810	0.147	0.00000337	*RARB*	0.003
	rs715500178	28	29,695,289	0.474	0.00000165	*NDST2*	0.044
	rs715500178	28	29,695,289	0.474	0.00000165	*ZSWIM8*	0.048
	rs720113004	29	34,597,655	0.039	0.00000049	*NTM*	0.045
	rs520498826	29	49,201,864	0.894	0.00000250	*CD81*	0.009

BTA^1^ – *Bos taurus* autosome, Position^2^ – basis pair location of the signicant genetic marker; MAF^3^ – minor frequency allele; P-value^4^ – significance value obtained in GWAS analysis; Adjusted p-value^5^ – adjusted p-value obtained by gene prioritization

**Table 3:** Prioritized candidate gene for scrotal circumference, post-weight gain, and yearling weight identified by Guildify and ToppGene analysis

Coefficient	SNP	BTA^1^	Position^2^	MAF^3^	*P*-value^4^	Gene	Adjusted *P*-value^5^
Scrotal circumference							
Intercept	rs461649851	2	31,692,781	0.551	0.00000008	*GRB14*	0.008
	rs444130700	3	30,792,574	0.357	0.00000149	*WNT2B*	0.048
	rs439617979	10	49,596,492	0.144	0.00000256	*RORA*	0.006
	rs133204123	10	59,132,956	0.226	0.00000021	*CYP19A1*	< 0.0000001
	rs473452904	14	23,216,603	0.803	0.0000005	*LYN*	< 0.0000001
	rs211490057	16	57,727,044	0.491	0.00000098	*PAPPA2*	0.016
Slope	rs461649851	2	31,692,781	0.551	0.00000323	*GRB14*	0.008
	rs717748518	10	51,018,655	0.089	0.0000027	*MYO1E*	0.037
	rs133204123	10	59,132,956	0.226	0.00000287	*CYP19A1*	< 0.0000001
	rs473452904	14	23,216,603	0.803	0.00000004	*LYN*	< 0.0000001
	rs524764569	16	57,807,034	0.720	0.000001	*PAPPA2*	0.016
	rs471174155	23	17,123,734	0.801	0.00000088	*POLH*	0.008
Post-weaning weight gain							
Slope	rs520046194	13	71,535,726	0.177	4.50E-07	*PTPRT*	0.008
Yearling weight							
Intercept	rs210000614	14	19,427,775	0.100	0.0000000	*PRKDC*	0.009
	rs521230847	14	23,140,778	0.502	0.0000001	*LYN*	0.001
Slope	rs210000614	14	19,427,775	0.100	0.0000001	*PRKDC*	0.008
	rs521230847	14	23,140,778	0.502	0.0000008	*LYN*	0.002
	rs211065097	23	9,306,293	0.155	0.0000020	*PPARD*	0.008

BTA^1^ – *Bos taurus* autosome, Position^2^ – basis pair location of the signicant genetic marker; MAF^3^ – minor frequency allele; P-value^4^ – significance value obtained in GWAS analysis; Adjusted p-value^5^ – adjusted p-value obtained by gene prioritization

## Discussion

We performed a GWAS via ssGRN to detect candidate genomic regions associated with sexual precocity indicators (AFC and SC) and growth traits (YW and PWG) (Figs. 1, 2, 3 and 4). Some identified genomic regions are common between slopes and intercepts and between traits. The SNP markers detected (− log_10_(p-value) > 5.45) showed reranking across EG levels, in which the effects on the Low EG levels were different from those on the High EG levels (Fig. 5). The SNP effects changed in magnitude and direction according to the EG level. Several studies of reproductive traits in dairy cattle [31] and beef cattle [3, 12] and reproduction, body composition, and growth traits in pigs [32] have shown that different environmental conditions can cause substantial changes in SNP effect estimates.

### Genomic regions for RN coefficients affecting AFC

The GWAS analysis for AFC has identified 33 and 32 significant SNP markers associated with the intercept and slope, respectively. These markers map 29 genes that are shared between them (Table 2), which explains the high correlation between the coefficients of the reaction norm, which was r_g_ = 0.93 [4]. Candidate genes with significant effect (${-\text{l}\text{o}\text{g}}_{10}\left(\text{p}-\text{v}\text{a}\text{l}\text{u}\text{e}\right)>5.45$) on the AFC intercept and slope were related to lipid metabolism. The *PLCB1* on BTA13 encodes a phospholipase and is related to the hydrolysis of phospholipids into fatty acids [33] and to the energy metabolism process [34]. In addition, it was associated with carcass fat deposition in cattle [35]. This gene is essential for fertilization in mammals since it is widely distributed on the oocyte plasma membrane and, independently, is involved in sperm–oocyte fusion as an extracellular component in mouse oocytes [36]. Additionally, it is expressed in bovine oocytes during early growth and meiotic maturation and appears to be required for successful sperm–oocyte interactions during fertilization [37, 38]. The *PLCB1* gene has previously been associated with heat stress in sheep and goats [39], cattle [40, 41], and catfish [34], suggesting that it can be an indicator of the GxE interaction response. The *CTSH* (BTA21) is a gene belonging to the cathepsin family and is involved in adipocyte differentiation [42]. The age at first calving, a trait related to female sexual precocity, can be affected by the level of subcutaneous fat in cattle [43]. These findings indicate that both genes (*PLCB1 and CTSH)* have pleiotropic properties, supporting the occurrence of a favorable effect on subcutaneous fat deposition and precocity/fertility traits in bovine females [44–46].

The *FUT8* gene on BTA10 encodes an enzyme that transfers fucose from GDP-fucose to glycoconjugates such as glycoproteins [47]. This gene was also associated with AFC [48] and sire conception rate [49]. Deletion of this gene in mice induced severe growth retardation and death during postnatal development [50]. Furthermore, *FUT8* is an essential gene for maintaining normal physiological homeostasis [47, 50, 51], suggesting its role in adapting to environmental variations. The *PPP1R12A* gene (BTA5) is involved in insulin signaling regulation [52] and is associated with Nellore female sexual precocity [12]. This gene is promising since metabolic homeostasis mediated by insulin and glucose has an important role in the nervous system and ovary [53]. *FGF10* (BTA20) is a member of the fibroblast growth factor family and is of particular interest for livestock reproduction because it is expressed in theca cells, luteal cells, and oocytes [54, 55] in addition to playing an important role in oocyte maturation in bovines [56–58].

The functional enrichment analysis identified the major biological processes related to the positive regulation of cell communication (GO:0010647), neuropeptide catabolic process (GO:0010813), positive regulation of signaling (GO:0023056), MAPK cascade (GO:0000165), myoblast fusion involved in skeletal muscle regeneration (GO:0014905) and molecular function in lipid binding (GO:0008289, Table 4). These biological processes affect AFC by improving signaling pathways that involve hormones like estrogen and testosterone (GO:0010647 and GO:0023056), but also by hormones that affect cellular processes, such as growth, differentiation, and hormonal activities (GO:0000165) and early muscle development (GO:0014905, Table 4) in response to hormonal changes associated with early puberty [46, 59].

**Table 4:** Gene ontology enrichment analysis for biological process (BP) and molecular function (MF) of the genes identified for age at first calving (AFC), scrotal circumference (SC), and yearling weight (YW) [for more details, see Additional file 1: Table S7 to S9]

Trait	Ontology	ID	Description	*p*-value	q-value	Gene
AFC	BP	GO:0002456	T cell mediated immunity	0.00051	0.02905	CTSH/CD81
	BP	GO:0010647	positive regulation of cell communication	0.00106	0.02905	SYT1/CTSH/PLCB1/CD81
	BP	GO:0023056	positive regulation of signaling	0.00107	0.02905	SYT1/CTSH/PLCB1/CD81
	BP	GO:0000165	MAPK cascade	0.00167	0.02905	CTSH/PLCB1/CD81
	BP	GO:0010813	neuropeptide catabolic process	0.00177	0.02905	CTSH
	BP	GO:0014905	myoblast fusion involved in skeletal muscle regeneration	0.00354	0.03138	CD81
	MF	GO:0008289	lipid binding	0.00157	0.00565	PLCB1/CD81/DYSF
SC	MF	GO:0070330	aromatase activity	0.00206	0.00378	CYP19A1
	MF	GO:0016712	oxidoreductase activity, acting on paired donors, with incorporation or reduction of molecular oxygen, reduced flavin or flavoprotein as one donor, and incorporation of one atom of oxygen	0.0036	0.00378	CYP19A1
	MF	GO:0005506	iron ion binding	0.023	0.00692	CYP19A1
	MF	GO:0016705	oxidoreductase activity, acting on paired donors, with incorporation or reduction of molecular oxygen	0.023	0.00692	CYP19A1
YW	BP	GO:0046838	phosphorylated carbohydrate dephosphorylation	0.00354	0.02507	BPNT2
	BP	GO:0046855	inositol phosphate dephosphorylation	0.00354	0.02507	BPNT2
	BP	GO:0071545	inositol phosphate catabolic process	0.00442	0.02507	BPNT2
	BP	GO:0046854	phosphatidylinositol phosphate biosynthetic process	0.00706	0.02507	BPNT2
	BP	GO:0043647	inositol phosphate metabolic process	0.00794	0.02507	BPNT2
	BP	GO:0006334	nucleosome assembly	0.01583	0.0375	NAP1L5
	BP	GO:0006661	phosphatidylinositol biosynthetic process	0.0202	0.03779	BPNT2
	BP	GO:0034728	nucleosome organization	0.02194	0.03779	NAP1L5
	BP	GO:0046488	phosphatidylinositol metabolic process	0.02975	0.04026	BPNT2
	BP	GO:0008654	phospholipid biosynthetic process	0.04779	0.04765	BPNT2
	BP	GO:0045017	glycerolipid biosynthetic process	0.05204	0.04931	BPNT2
	MF	GO:0008252	nucleotidase activity	0.00308	0.00324	BPNT2
	MF	GO:0016791	phosphatase activity	0.04556	0.03414	BPNT2
	MF	GO:0042578	phosphoric ester hydrolase activity	0.06487	0.03414	BPNT2

The MAPK signaling pathway interacts with different intracellular signaling pathways, such as steroid receptors that influence uterine cell proliferation [60], and plays a key role in embryonic and yolk sac angiogenesis during fetal-placental development [61]. Furthermore, evidence shows that MAPK cascades are involved in several male reproductive processes such as spermatogenesis, sperm maturation, sperm capacitation, and acrosome reaction before oocyte fertilization [62]. In livestock species, Gonçalves et al. [63] found differentially expressed genes involved in the MAPK pathway in the cervix at different stages of the estrous cycle in sheep and cattle. The enriched genes were also involved in several immune system processes (see additional Table S7), such as the regulation of adaptive immune memory response (GO:0090716; GO:1,905,674; GO:1,905,676), processes associated with T cells (GO:0002456; GO:0035783; GO:2,001,188; GO:2,001,190; GO:0035739; GO:2,000,561; GO:2,000,563), regulation of B cell receptor (GO:0050855; GO:0050861), and interleukins (GO:0035722; GO:0070498; GO:0071349). The immune and reproductive systems closely interact due to the sharing of certain cytokines and their receptors, which can affect neuroendocrine events, ovarian function, placenta, and embryo development and may play a role in immunological reproductive failure [64]. In Holstein cattle, Thompson-Crispi et al. [65] reported favorable genetic associations between the adaptive immune response and reproductive traits, suggesting that selection for overall immune responsiveness may lead to a positive response in reproductive traits in cattle.

### Genomic regions for RN coefficients affecting SC

Multiple prioritized genes (*GRB14*, *CYP19A1*, *LYN*, and *PAPPA2*) were associated with both SC reaction norm coefficients (Table 3). The *GRB14* gene, on BTA2, encodes a growth factor receptor-binding protein, and mRNA molecules of this gene have been found to be expressed at high levels in the mammalian ovary, liver, kidney, and skeletal muscle [66, 67]. In addition, Bohrer et al. [68] showed that *GRB14* mRNA is expressed in granulosa and theca cells during different stages of follicular development, suggesting that this gene may play a regulatory role during follicular divergence in cattle. The *PAPPA2* gene, located on BTA16, affects reproduction and fertility and has important roles in pregnancy and postnatal growth [69]. SNP markers within the *PAPPA2* gene have been associated with calving ease and productive life in Holstein cattle, playing an important role in the breeding of first-calf heifers and affecting essential reproductive aspects such as calving interval, days to calving, and pregnancy rate [70]. These results suggest a pleiotropic effect of genes that influence both SC and female sexual performance traits, corroborating studies reporting favorable genetic correlation estimates between these traits [44, 71–73].

The *RORA* gene on BTA10, associated only with the SC intercept coefficient, encodes a nuclear receptor that is essential for the activation of myogenic-specific markers and regulates several genes involved in lipid metabolism [74, 75]. Moreover, it is related to steroid hormone receptor activity and, when combined with this hormone, produces the signal within the cell to initiate a change in cell activity or function [76]. Additionally, associated only with SC intercept, the *WNT2B* gene encodes a member of the Wnt family of secreted and highly conserved signaling factors that function in a variety of developmental processes, including the regulation of cell growth and differentiation [77, 78]. Using RNA-seq technology, Zhang et al. [79] identified a cluster of transcripts, including *WNT2B* mRNA, that may have direct or indirect functions in the initiation of puberty in sheep, which may provide new insights into the mechanisms that trigger puberty in ruminant species. In cattle, Liu et al. [80] reported that the *WNT2B* gene was enriched in male gonad development, supporting the influence of this gene on scrotal circumference. The *MYO1E* gene (BTA10) was associated with slope and is a structural myofibrillar protein related to the response of plants to recovery growth. Myogenic factors are associated with endocrine factors, which play important roles in the regulation of muscle mass, fiber size, nutrient partitioning, and reproduction [81]. This gene is also associated with the rapid differentiation of neonatal epithelial cells into mature intestinal epithelial cells (Benesh et al., 2010) and with feed efficiency in chickens [82].

The GO terms for SC (see additional Table S8) indicated that the *CYP19A1* gene was associated with oxidoreductase activity (GO:0016712 and GO:0016705), aromatase activity (GO:0070330) and iron ion binding (GO:0005506). The *CYP19A1* gene, enriched for aromatase activity, is mainly expressed in Leydig and testicular germ cells [83, 84] and encodes a member of the cytochrome P450 superfamily of enzymes. Cytochrome P450 aromatase is an enzyme that catalyzes the conversion of androgens, such as testosterone, to estrogens, which act as sex steroid hormones but also function during growth and differentiation [85]. These enzymes are highly expressed in both the gonads and the brain in humans [86]. Variation in the *CYP19A1* gene was associated with growth and reproduction in mice and humans [87]. Using RNA-seq to profile the testicular transcriptome in premature and mature sheep, Yang et al. [88] observed that *CYP19A1* expression levels significantly increased with animals’ age, indicating that this gene may play an important role in ruminants’ testicular development.

### Genomic regions for RN coefficients affecting PWG

For PWG, only the gene *PTPRT* gene on BTA13 (FDR-corrected p-value < 0.05) was detected in the prioritization analysis for the slope coefficient (Table 3). The *PTPRT* gene on BTA13 encodes a protein from the tyrosine phosphatase (PTP) family, related to a variety of physiological processes, including cell growth, differentiation, metabolism, cell cycle regulation, and cytoskeletal function [89]. In production animals, a relationship between the *PTPRT* gene polymorphisms and resistance to some bacterial and parasitic infections was observed, such as resistance to brucellosis in goats [90] and tuberculosis in cattle [91]. In this sense, the fact that this gene is associated with resistance to different infections lays the groundwork for potential GxE interaction. Furthermore, in a genomic association study, the *PTPRT* gene was shown to be associated with birth weight in ovine [92], elucidating the importance of this gene in growth traits.

### Genomic regions for RN coefficients affecting YW

For YW, BTA14 had a major influence on this trait, and two prioritized genes (*LYN* and *PRKDC*) are associated with both reaction norm coefficients. The *LYN* gene encodes a Src family kinase that is involved in cell proliferation, survival, differentiation, migration, adhesion, and apoptosis [41, 93]. In beef cattle, this gene has been associated with sexual precocity in heifers [12, 46], growth [94], feed intake [95], carcass [96], and meat quality traits [96]. In addition, this gene was also associated with SC in this study. It is important to mention that the *LYN* gene is located within a promising QTL on BTA14 that harbors a variety of genes influencing a wide range of traits of economic interest in livestock [97, 98]. *PRKDC*, also known as *XRCC7*, is related to embryonic development, interferon tau expression, and the trophoblast development rate in cattle [58]. In other farm species, this gene has been associated with body size in sheep [99] and feed conversion efficiency in pigs [100], suggesting that this gene plays an important role in growth and development. Although Guildify did not identify the PLAG1 gene on BTA14 during gene prioritization, this gene has a striking effect on biological mechanisms that might help explain the variability in body weight and adaptability to environmental conditions. The SNP markers identified in the BTA14 region were 20.58–25.11 Mb (*LYN, TMEM68, PLAG1, CHCHD7*, and *MOS*), affecting the MAPK signaling pathway and affecting cell proliferation and growth by mediating IGF-1 and − 2, which control the energy metabolism linked to tissue development [101]. In multiple breeds, Utsunomiya et al. [102] studied Nellore cattle, and Bouwman et al. [103] reported that specific haplotypes associated with the *PLAG1* mutation have positive effects on weight and conformation traits.

The *PPARD* on BTA23, associated with the YW slope, encodes the peroxisome proliferator-activated receptor delta, a transcription factor predominantly expressed in skeletal muscle [104] involved in the development, lipid metabolism, energy expenditure, tissue repair and regeneration, and inflammation [105]. *PPARD* acts as a key regulator of energy metabolism in skeletal muscle, using lipids as the main energy substrate [106], thus allowing glucose to become more available for other physiological processes [105]. In dairy cows, this gene was implicated in muscle fatty acid transport and oxidation during early lactation [107] and influences factors such as lactation onset and lipid supply [108, 109]. The enrichment analysis for YW (see additional Table S9) identified potential candidate genes (*BPNT2* and *NAP1L5*) involved in processes related to phosphorylated carbohydrate dephosphorylation (GO:0046838), inositol phosphate (GO:0046855, GO:0071545 and GO:0043647), phosphatidylinositol (GO:0046854, GO:0006661 and GO:0046488), nucleosome (GO:0006334 and GO:0034728), phospholipid (GO:0008654), glycerolipid (GO:0045017), nucleotidase activity (GO:0008252), phosphatase activity (GO:0016791), phosphoric ester and hydrolase activity (GO:0042578). Most of the enriched GO terms are involved in phosphate metabolism. Phosphate plays essential roles in diverse cellular actions, such as energy metabolism, differentiation, proliferation, and specific functions of differentiated cells [110], all of which are crucial for the growth and development of organisms. In addition, inositol phosphates are related to energy homeostasis, antioxidant and anti-inflammatory activities, and play a role as neurotransmitters [111]. There is evidence that inositol mimics the insulin signaling pathway [112]. In this sense, Lee & Bedford [113] suggested that possibly inositol induces glucose uptake, leading to an increased energy supply in skeletal muscle to support growth, providing insights into potential inositol mechanisms in promoting the animal growth response.

## Conclusions

GWAS via reaction norm detected candidate genes affecting both the intercept and slope on EG for sexual precocity indicator (AFC and SC) and growth (YW and PWG) traits related to several biological mechanisms by which beef cattle respond to environmental changes. The genes found have been previously associated with growth, adaptative and reproductive traits in cattle and other livestock species. In general, the potential candidate genes identified were involved in several biological mechanisms related to lipid metabolism, immune response, MAPK signaling pathway, and energy and phosphate metabolism. The results of the GWAS analysis provide a better understanding of the underlying biological processes associated with growth and reproductive traits in Nellore cattle raised under different environmental conditions.

### Electronic supplementary material

Below is the link to the electronic supplementary material.

Supplementary Material 1

Supplementary Material 2

## Data Availability

The phenotypic and genotypic information are available for academic use from the authors upon reasonable request (contacting the researcher Lucia Galvão de Albuqueruqe to e-mail: galvao.albuquerque@unesp.br) and with permission of Alliance Nellore breeding program (https://gensys.com.br).
