# Supplementary material for: Genome-wide association study considering genotype-by-environment interaction for productive and reproductive traits using whole-genome sequencing in Nellore cattle
Source: BMC Genomics. 2024 Jun 20;25:623. doi: 10.1186/s12864-024-10520-x (PMC11188527; doi:10.1186/s12864-024-10520-x)
Supplement: Supplementary file 1 — Supplementary Material 1 [file 12864_2024_10520_MOESM1_ESM.docx]

# Additional file 1

**Table S1** Average distance between SNPs in each chromosome in base pair (bp).

| Chromosome | Trait^1^ | | | |
| --- | --- | --- | --- | --- |
|  | AFC | SC | PWG | YW |
| 1 | 495.4 | 1189.1 | 1186.8 | 1184.2 |
| 2 | 527.1 | 1275.4 | 1274.5 | 1271.0 |
| 3 | 505.7 | 1170.8 | 1169.5 | 1166.1 |
| 4 | 456.7 | 1077.2 | 1076.5 | 1073.5 |
| 5 | 572.7 | 1413.0 | 1412.2 | 1407.8 |
| 6 | 553.7 | 1352.4 | 1350.0 | 1346.9 |
| 7 | 500.7 | 1186.5 | 1185.1 | 1182.0 |
| 8 | 533.6 | 1302.7 | 1302.0 | 1297.1 |
| 9 | 503.7 | 1210.2 | 1210.0 | 1206.5 |
| 10 | 467.9 | 1045.7 | 1044.5 | 1041.8 |
| 11 | 494.7 | 1142.3 | 1140.8 | 1138.8 |
| 12 | 467.4 | 1110.1 | 1108.6 | 1105.4 |
| 13 | 501.6 | 1174.3 | 1173.7 | 1170.0 |
| 14 | 513.0 | 1240.0 | 1240.3 | 1235.8 |
| 15 | 448.5 | 1051.2 | 1050.8 | 1047.8 |
| 16 | 491.2 | 1209.0 | 1206.8 | 1203.7 |
| 17 | 431.4 | 993.6 | 991.5 | 989.3 |
| 18 | 454.3 | 1006.7 | 1004.7 | 1001.7 |
| 19 | 488.2 | 1151.8 | 1151.5 | 1148.4 |
| 20 | 494.8 | 1232.8 | 1232.0 | 1227.2 |
| 21 | 461.4 | 1094.6 | 1094.7 | 1091.4 |
| 22 | 474.9 | 1104.8 | 1102.8 | 1100.4 |
| 23 | 394.9 | 563.7 | 557.4 | 554.4 |
| 24 | 514.0 | 643.7 | 645.6 | 645.3 |
| 25 | 490.0 | 581.9 | 584.0 | 583.6 |
| 26 | 499.4 | 611.5 | 613.7 | 613.5 |
| 27 | 471.2 | 578.3 | 581.3 | 581.5 |
| 28 | 471.1 | 580.4 | 582.1 | 582.3 |
| 29 | 488.7 | 598.7 | 601.6 | 601.3 |
| mean | 488.6 | 1030.8 | 1030.2 | 1027.5 |

^1^AFC: age at first calving; SC: scrotal circumference; PWG: post-weaning gain; and YW: yearling weight.

**Table S2** Relevant keywords used in Guildify.

| Trait | Keyword |
| --- | --- |
| Age at First Calving | Maturity, puberty, growth, weight, score, fertility, ovary, oocyte, progesterone. |
| Scrotal Circumference | Scrotal, testicular, testes, testis, sperm, semen, spermatozoa, spermatogenesis, fertility. |
| Post-Weaning Weight Gain | Weight gain, Growth rate, Feed efficiency, performance, Muscle, Metabolism, Carcass, protein. |
| Yearling Weight | Weight gain, Growth rate, Feed efficiency, performance, Muscle, Metabolism, Carcass, protein. |

**Table S3** Summary of significant SNPs detected by GWAS for reaction norm coefficients of intercept and slope for age at first calving (AFC) in Nellore cattle.

| s | SNP | Chromosome | Position (bp) | MAF | p-value | Gene |
| --- | --- | --- | --- | --- | --- | --- |
| Intercept | rs43230433 | 1 | 44063185 | 0.035 | 0.00000113 | *COL8A1* |
|  | rs210170805 | 1 | 145031416 | 0.058 | 0.00000121 | *ADARB1* |
|  | rs381434568 | 1 | 150106131 | 0.516 | 0.00000265 | *KCNJ6* |
|  | rs110470442 | 3 | 8634391 | 0.756 | 0.00000056 | *ITLN2* |
|  | rs135363381 | 3 | 21540509 | 0.876 | 0.00000051 | *RNF115* |
|  | rs208220012 | 3 | 22101652 | 0.070 | 0.00000017 | *BCL9* |
|  | rs208048961 | 3 | 26122562 | 0.277 | 0.00000188 | *TTF2* |
|  | rs380631872 | 3 | 29686891 | 0.503 | 0.00000102 | *PHTF1* |
|  | rs716063888 | 3 | 30747674 | 0.579 | 0.00000321 | *ST7L* |
|  | rs134647973 | 3 | 78315194 | 0.344 | 0.00000295 | *SLC35D1* |
|  | rs716562586 | 5 | 4998229 | 0.112 | 0.00000126 | *GLIPR1* |
|  | rs379055283 | 5 | 9003285 | 0.653 | 0.00000167 | *SYT1* |
|  | rs720484023 | 5 | 9385330 | 0.278 | 0.00000167 | *PPP1R12A* |
|  | rs715728654 | 7 | 55339011 | 0.424 | 0.00000145 | *KCTD16* |
|  | rs721185716 | 8 | 60555086 | 0.080 | 0.00000178 | *SNORA70* |
|  | rs517426012 | 8 | 69530246 | 0.335 | 0.00000267 | *BMP1* |
|  | rs517426012 | 8 | 69530246 | 0.335 | 0.00000267 | *PHYHIP* |
|  | rs466005125 | 9 | 86187067 | 0.444 | 0.00000189 | *UST* |
|  | rs445538954 | 10 | 77658449 | 0.537 | 0.00000233 | *FUT8* |
|  | rs519726335 | 11 | 614526 | 0.218 | 0.00000027 | *ANAPC1* |
|  | rs519493432 | 11 | 12923464 | 0.034 | 0.00000111 | *DYSF* |
|  | rs136849909 | 13 | 1263836 | 0.878 | 0.00000136 | *PLCB1* |
|  | rs523777757 | 13 | 59836380 | 0.334 | 0.00000147 | *SNPH* |
|  | rs523777757 | 13 | 59836380 | 0.334 | 0.00000147 | *bta-mir-2285bu-1* |
|  | rs208954324 | 14 | 19491973 | 0.825 | 0.00000238 | *PRKDC* |
|  | rs41734596 | 14 | 28169018 | 0.863 | 0.00000044 | *YTHDF3* |
|  | rs520454646 | 14 | 29257049 | 0.388 | 0.00000085 | *CYP7B1* |
|  | rs723811441 | 14 | 31027180 | 0.777 | 0.00000256 | *SGK3* |
|  | rs43213508 | 14 | 31540197 | 0.075 | 0.00000114 | *CPA6* |
|  | rs43195263 | 14 | 32141429 | 0.863 | 0.00000259 | *PREX2* |
|  | rs133920327 | 14 | 76084780 | 0.177 | 0.00000182 | *CNGB3* |
|  | rs208915092 | 16 | 61611420 | 0.979 | 0.00000060 | *ACBD6* |
|  | rs521155455 | 18 | 10690957 | 0.243 | 0.00000163 | *MEAK7* |
|  | rs210107085 | 18 | 61235843 | 0.248 | 0.00000278 | *MGC138914* |
|  | rs134265088 | 19 | 38330461 | 0.856 | 0.00000002 | *SKAP1* |
|  | rs456261266 | 20 | 30542035 | 0.050 | 0.00000138 | *FGF10* |
|  | rs720222086 | 20 | 37304855 | 0.823 | 0.00000313 | *NIPBL* |
|  | rs447539658 | 21 | 25173850 | 0.905 | 0.00000344 | *MORF4L1* |
|  | rs380578567 | 21 | 25211390 | 0.046 | 0.00000030 | *CTSH* |
|  | rs210224020 | 21 | 26556144 | 0.821 | 0.00000025 | *ABHD17C* |
|  | rs475843527 | 21 | 26731693 | 0.546 | 0.00000025 | *CEMIP* |
|  | rs723392301 | 23 | 20241067 | 0.050 | 0.00000207 | *ADGRF1* |
|  | rs477627963 | 23 | 29261939 | 0.042 | 0.00000016 | *OR12D2F* |
|  | rs110296566 | 24 | 19710489 | 0.026 | 0.00000079 | *CELF4* |
|  | rs519423782 | 24 | 57664815 | 0.063 | 0.00000283 | *ALPK2* |
|  | rs464263309 | 25 | 33683982 | 0.366 | 0.00000014 | *BAZ1B* |
|  | rs526123324 | 25 | 38124298 | 0.379 | 0.00000058 | *bta-mir-12052* |
|  | rs526123324 | 25 | 38124298 | 0.379 | 0.00000058 | *USP42* |
|  | rs42102555 | 26 | 46691693 | 0.132 | 0.00000332 | *DOCK1* |
|  | rs211337716 | 27 | 2647914 | 0.661 | 0.00000322 | *CSMD1* |
|  | rs714730097 | 27 | 7469393 | 0.170 | 0.00000350 | *GPM6A* |
|  | rs453588982 | 27 | 40147406 | 0.836 | 0.00000197 | *TOP2B* |
|  | rs382211336 | 27 | 40216810 | 0.147 | 0.00000337 | *RARB* |
|  | rs523189830 | 28 | 9425241 | 0.347 | 0.00000080 | *MTR* |
|  | rs715500178 | 28 | 29695289 | 0.474 | 0.00000168 | *ZSWIM8* |
|  | rs715500178 | 28 | 29695289 | 0.474 | 0.00000168 | *NDST2* |
|  | rs720113004 | 29 | 34597655 | 0.039 | 0.00000050 | *NTM* |
|  | rs520498826 | 29 | 49201864 | 0.894 | 0.00000259 | *CD81* |
| Slope | rs43230433 | 1 | 44063185 | 0.035 | 0.00000127 | *COL8A1* |
|  | rs210170805 | 1 | 145031416 | 0.058 | 0.00000129 | *ADARB1* |
|  | rs381434568 | 1 | 150106131 | 0.516 | 0.00000314 | *KCNJ6* |
|  | rs110470442 | 3 | 8634391 | 0.756 | 0.00000064 | *ITLN2* |
|  | rs135363381 | 3 | 21540509 | 0.976 | 0.00000050 | *RNF115* |
|  | rs208220012 | 3 | 22101652 | 0.070 | 0.00000022 | *BCL9* |
|  | rs208048961 | 3 | 26122562 | 0.277 | 0.00000242 | *TTF2* |
|  | rs380631872 | 3 | 29686891 | 0.503 | 0.00000125 | *PHTF1* |
|  | rs134647973 | 3 | 78315194 | 0.344 | 0.00000287 | *SLC35D1* |
|  | rs716562586 | 5 | 4998229 | 0.112 | 0.00000122 | *GLIPR1* |
|  | rs379055283 | 5 | 9003285 | 0.653 | 0.00000181 | *SYT1* |
|  | rs720484023 | 5 | 9385330 | 0.278 | 0.00000219 | *PPP1R12A* |
|  | rs715728654 | 7 | 55339011 | 0.424 | 0.00000182 | *KCTD16* |
|  | rs721185716 | 8 | 60555086 | 0.080 | 0.00000194 | *SNORA70* |
|  | rs517426012 | 8 | 69530246 | 0.335 | 0.00000262 | *BMP1* |
|  | rs517426012 | 8 | 69530246 | 0.335 | 0.00000262 | *PHYHIP* |
|  | rs466005125 | 9 | 86187067 | 0.444 | 0.00000183 | *UST* |
|  | rs445538954 | 10 | 77658449 | 0.537 | 0.00000266 | *FUT8* |
|  | rs519726335 | 11 | 614526 | 0.218 | 0.00000030 | *ANAPC1* |
|  | rs519493432 | 11 | 12923464 | 0.034 | 0.00000112 | *DYSF* |
|  | rs136849909 | 13 | 1263836 | 0.978 | 0.00000137 | *PLCB1* |
|  | rs523777757 | 13 | 59836380 | 0.334 | 0.00000142 | *SNPH* |
|  | rs523777757 | 13 | 59836380 | 0.334 | 0.00000142 | *bta-mir-2285bu-1* |
|  | rs208954324 | 14 | 19491973 | 0.825 | 0.00000244 | *PRKDC* |
|  | rs41734596 | 14 | 28169018 | 0.919 | 0.00000050 | *YTHDF3* |
|  | rs520454646 | 14 | 29257049 | 0.388 | 0.00000109 | *CYP7B1* |
|  | rs723811441 | 14 | 31027180 | 0.777 | 0.00000264 | *SGK3* |
|  | rs43213508 | 14 | 31540197 | 0.075 | 0.00000119 | *CPA6* |
|  | rs43195263 | 14 | 32141429 | 0.863 | 0.00000267 | *PREX2* |
|  | rs133920327 | 14 | 76084780 | 0.177 | 0.00000219 | *CNGB3* |
|  | rs208915092 | 16 | 61611420 | 0.979 | 0.00000066 | *ACBD6* |
|  | rs521155455 | 18 | 10690957 | 0.243 | 0.00000160 | *MEAK7* |
|  | rs134265088 | 19 | 38330461 | 0.856 | 0.00000002 | *SKAP1* |
|  | rs456261266 | 20 | 30542035 | 0.050 | 0.00000175 | *FGF10* |
|  | rs380578567 | 21 | 25211390 | 0.046 | 0.00000032 | *CTSH* |
|  | rs210224020 | 21 | 26556144 | 0.821 | 0.00000028 | *ABHD17C* |
|  | rs475843527 | 21 | 26731693 | 0.546 | 0.00000027 | *CEMIP* |
|  | rs723392301 | 23 | 20241067 | 0.050 | 0.00000240 | *ADGRF1* |
|  | rs477627963 | 23 | 29261939 | 0.042 | 0.00000022 | *OR12D2F* |
|  | rs110296566 | 24 | 19710489 | 0.026 | 0.00000081 | *CELF4* |
|  | rs519423782 | 24 | 57664815 | 0.063 | 0.00000305 | *ALPK2* |
|  | rs464263309 | 25 | 33683982 | 0.366 | 0.00000015 | *BAZ1B* |
|  | rs526123324 | 25 | 38124298 | 0.379 | 0.00000062 | *bta-mir-12052* |
|  | rs526123324 | 25 | 38124298 | 0.379 | 0.00000062 | *USP42* |
|  | rs453588982 | 27 | 40147406 | 0.836 | 0.00000202 | *TOP2B* |
|  | rs382211336 | 27 | 40216810 | 0.147 | 0.00000337 | *RARB* |
|  | rs523189830 | 28 | 9425241 | 0.347 | 0.00000089 | *MTR* |
|  | rs715500178 | 28 | 29695289 | 0.474 | 0.00000165 | *NDST2* |
|  | rs715500178 | 28 | 29695289 | 0.474 | 0.00000165 | *ZSWIM8* |
|  | rs720113004 | 29 | 34597655 | 0.039 | 0.00000049 | *NTM* |
|  | rs520498826 | 29 | 49201864 | 0.894 | 0.00000250 | *CD81* |

MAF – minor frequency allele

**Table S4** Summary of significant SNPs detected by GWAS for reaction norm coefficients of intercept and slope for scrotal circumference (SC) in Nellore cattle.

| Coefficient | SNP | BTA | Position (bp) | MAF | P-value | Gene |
| --- | --- | --- | --- | --- | --- | --- |
| Intercept | rs461649851 | 2 | 31692781 | 0.551 | 0.00000008 | *GRB14* |
|  | rs444130700 | 3 | 30792574 | 0.357 | 0.00000149 | *WNT2B* |
|  | rs444130700 | 3 | 30792574 | 0.357 | 0.00000149 | *ST7L* |
|  | rs439617979 | 10 | 49596492 | 0.144 | 0.00000256 | *RORA* |
|  | rs133204123 | 10 | 59132956 | 0.226 | 0.00000021 | *CYP19A1* |
|  | rs473452904 | 14 | 23216603 | 0.803 | 0.00000050 | *LYN* |
|  | rs211490057 | 16 | 57727044 | 0.491 | 0.00000098 | *PAPPA2* |
|  | rs210784233 | 23 | 17176660 | 0.746 | 0.00000000 | *MRPS18A* |
|  | rs210784233 | 23 | 17176660 | 0.746 | 0.00000000 | *RSPH9* |
| Slope | rs461649851 | 2 | 31692781 | 0.551 | 0.00000323 | *GRB14* |
|  | rs717312147 | 3 | 1.12E+08 | 0.031 | 0.00000210 | *CSMD2* |
|  | rs717748518 | 10 | 51018655 | 0.089 | 0.00000270 | *MYO1E* |
|  | rs133204123 | 10 | 59132956 | 0.226 | 0.00000287 | *CYP19A1* |
|  | rs473452904 | 14 | 23216603 | 0.803 | 0.00000004 | *LYN* |
|  | rs524764569 | 16 | 57807034 | 0.720 | 0.00000100 | *PAPPA2* |
|  | rs471174155 | 23 | 17123734 | 0.801 | 0.00000088 | *POLH* |
|  | rs471174155 | 23 | 17123734 | 0.801 | 0.00000088 | *XPO5* |
|  | rs210784233 | 23 | 17176660 | 0.746 | 0.00000009 | *RSPH9* |
|  | rs210784233 | 23 | 17176660 | 0.746 | 0.00000009 | *MRPS18A* |

BTA – *Bos taurus* autosome, Position – basis pair location of the significant genetic marker; MAF – minor frequency allele; P-value – significance value obtained in GWAS analysis

**Table S5** Summary of significant SNPs detected by GWAS for reaction norm coefficients of intercept and slope for post-weight gain (PWG) in Nellore cattle.

| Coefficient | SNP | BTA | Position | MAF | P-value | Gene |
| --- | --- | --- | --- | --- | --- | --- |
| Intercpt | rs109451695 | 6 | 36082149 | 0.027 | 2.50E-06 | *NAP1L5* |
|  | rs721075979 | 6 | 36919575 | 0.028 | 2.00E-06 | *HERC3* |
|  | rs110896026 | 6 | 37999825 | 0.842 | 2.30E-06 | *-* |
|  | rs520499681 | 25 | 5148200 | 0.570 | 1.00E-06 | *RBFOX1* |
|  | rs466005212 | 29 | 45651996 | 0.222 | 7.00E-07 | *-* |
| Slope | rs109451695 | 6 | 36082149 | 0.027 | 2.14E-06 | *HERC3* |
|  | rs109451695 | 6 | 36082149 | 0.027 | 2.14E-06 | *NAP1L5* |
|  | rs520046194 | 13 | 71535726 | 0.177 | 4.50E-07 | *PTPRT* |

BTA – *Bos taurus* autosome, Position – basis pair location of the significant genetic marker; MAF – minor frequency allele; P-value – significance value obtained in GWAS analysis

**Table S6** Summary of significant SNPs detected by GWAS for reaction norm coefficients of intercept and slope for yearling weight (YW) in Nellore cattle.

| Coefficient | SNP | BTA | Position | MAF | P-value | Gene |
| --- | --- | --- | --- | --- | --- | --- |
| Intercept | rs719752337 | 6 | 35773774 | 0.039 | 0.0000002 | *FAM13A* |
|  | rs520172865 | 6 | 36075516 | 0.829 | 0.0000002 | *HERC3* |
|  | rs109451695 | 6 | 36082149 | 0.029 | 0.000000001 | *NAP1L5* |
|  | rs723099741 | 6 | 37224559 | 0.026 | 0.0000004 | *FAM184B* |
|  | rs210865281 | 10 | 1E+08 | 0.489 | 0.0000024 | *PTPN21* |
|  | rs210122933 | 14 | 19246400 | 0.129 | 0.0000025 | *SPIDR* |
|  | rs210000614 | 14 | 19427775 | 0.199 | 0.0000000 | *PRKDC* |
|  | rs136337616 | 14 | 19986234 | 0.403 | 0.0000033 | *PPDPFL* |
|  | rs209777308 | 14 | 20581354 | 0.898 | 0.0000020 | *SNTG1* |
|  | rs109017499 | 14 | 21601179 | 0.803 | 0.0000001 | *U6* |
|  | rs723032570 | 14 | 22820699 | 0.875 | 0.0000001 | *XKR4* |
|  | rs109557968 | 14 | 23039018 | 0.827 | 0.0000003 | *TMEM68* |
|  | rs521230847 | 14 | 23140778 | 0.502 | 0.0000001 | *LYN* |
|  | rs453287612 | 14 | 23294566 | 0.751 | 0.000000001 | *U1* |
|  | rs453287612 | 14 | 23294566 | 0.751 | 0.000000001 | *MOS* |
|  | rs209373059 | 14 | 23376518 | 0.753 | 0.000000001 | *CHCHD7* |
|  | rs209373059 | 14 | 23376518 | 0.753 | 0.000000001 | *PLAG1* |
|  | rs137557469 | 14 | 23426105 | 0.481 | 0.000000001 | *SDR16C5* |
|  | rs382928906 | 14 | 23488260 | 0.129 | 0.000000001 | *SDR16C6* |
|  | rs208634699 | 14 | 23881038 | 0.890 | 0.000000001 | *BPNT2* |
|  | rs136548249 | 14 | 24408789 | 0.875 | 0.0000006 | *FAM110B* |
|  | rs1116439978 | 14 | 24593585 | 0.900 | 0.000000001 | *UBXN2B* |
|  | rs379987725 | 14 | 24823807 | 0.865 | 0.0000021 | *NSMAF* |
|  | rs208105290 | 14 | 25115153 | 0.895 | 0.0000012 | *TOX* |
| Slope | rs719752337 | 6 | 35773774 | 0.139 | 0.0000002 | *FAM13A* |
|  | rs520172865 | 6 | 36075516 | 0.929 | 0.0000001 | *HERC3* |
|  | rs109451695 | 6 | 36082149 | 0.128 | 0.000000001 | *NAP1L5* |
|  | rs723099741 | 6 | 37224559 | 0.225 | 0.0000013 | *FAM184B* |
|  | rs210865281 | 10 | 1E+08 | 0.488 | 0.0000014 | *PTPN21* |
|  | rs210122933 | 14 | 19246400 | 0.129 | 0.0000023 | *SPIDR* |
|  | rs210000614 | 14 | 19427775 | 0.099 | 0.0000001 | *PRKDC* |
|  | rs136337616 | 14 | 19986234 | 0.403 | 0.0000015 | *PPDPFL* |
|  | rs134659404 | 14 | 20463462 | 0.129 | 0.0000032 | *SNTG1* |
|  | rs207915962 | 14 | 21253926 | 0.480 | 0.0000015 | *ST18* |
|  | rs109017499 | 14 | 21601179 | 0.903 | 0.0000001 | *U6* |
|  | rs723032570 | 14 | 22820699 | 0.875 | 0.0000004 | *XKR4* |
|  | rs109557968 | 14 | 23039018 | 0.827 | 0.0000009 | *TMEM68* |
|  | rs521230847 | 14 | 23140778 | 0.502 | 0.0000008 | *LYN* |
|  | rs453287612 | 14 | 23294566 | 0.751 | 0.0000001 | *MOS* |
|  | rs453287612 | 14 | 23294566 | 0.751 | 0.0000001 | *U1* |
|  | rs209373059 | 14 | 23376518 | 0.753 | 0.000000001 | *CHCHD7* |
|  | rs209373059 | 14 | 23376518 | 0.753 | 0.000000001 | *PLAG1* |
|  | rs137557469 | 14 | 23426105 | 0.481 | 0.000000001 | *SDR16C5* |
|  | rs382928906 | 14 | 23488260 | 0.129 | 0.000000001 | *SDR16C6* |
|  | rs208634699 | 14 | 23881038 | 0.890 | 0.000000001 | *BPNT2* |
|  | rs136548249 | 14 | 24408789 | 0.875 | 0.0000027 | *FAM110B* |
|  | rs1116439978 | 14 | 24593585 | 0.900 | 0.00000001 | *UBXN2B* |
|  | rs379987725 | 14 | 24823807 | 0.865 | 0.0000026 | *NSMAF* |
|  | rs41621106 | 14 | 25159804 | 0.874 | 0.00000001 | *TOX* |
|  | rs483170311 | 23 | 8897028 | 0.470 | 0.0000034 | *ANKS1A* |
|  | rs483170311 | 23 | 8897028 | 0.470 | 0.0000034 | *TAF11* |
|  | rs211065097 | 23 | 9306293 | 0.155 | 0.0000020 | *PPARD* |
|  | rs718476723 | 25 | 25427100 | 0.047 | 0.0000030 | *GSG1L* |

BTA – *Bos taurus* autosome, Position – basis pair location of the significant genetic marker; MAF – minor frequency allele; P-value – significance value obtained in GWAS analysis

**Table S7**. Results of the Age at first calving enrichment analysis from the genes prioritized in the functional annotation study with whole-genome sequence data.

| Ontology^1^ | ID | Description | p-value | p-adjust | q-value | Gene |
| --- | --- | --- | --- | --- | --- | --- |
| BP | GO:0002456 | T cell mediated immunity | 0.000512 | 0.04582 | 0.029051 | *CTSH/CD81* |
| BP | GO:0010647 | positive regulation of cell communication | 0.00106 | 0.04582 | 0.029051 | *SYT1/CTSH/PLCB1/CD81* |
| BP | GO:0023056 | positive regulation of signaling | 0.001074 | 0.04582 | 0.029051 | *SYT1/CTSH/PLCB1/CD81* |
| BP | GO:0000165 | MAPK cascade | 0.001672 | 0.04582 | 0.029051 | *CTSH/PLCB1/CD81* |
| BP | GO:0010813 | neuropeptide catabolic process | 0.001769 | 0.04582 | 0.029051 | *CTSH* |
| BP | GO:0034238 | macrophage fusion | 0.001769 | 0.04582 | 0.029051 | *CD81* |
| BP | GO:0035783 | CD4-positive, alpha-beta T cell costimulation | 0.001769 | 0.04582 | 0.029051 | *CD81* |
| BP | GO:0043126 | regulation of 1-phosphatidylinositol 4-kinase activity | 0.001769 | 0.04582 | 0.029051 | *CD81* |
| BP | GO:0043128 | positive regulation of 1-phosphatidylinositol 4-kinase activity | 0.001769 | 0.04582 | 0.029051 | *CD81* |
| BP | GO:0046368 | GDP-L-fucose metabolic process | 0.001769 | 0.04582 | 0.029051 | *FUT8* |
| BP | GO:0048791 | calcium ion-regulated exocytosis of neurotransmitter | 0.001769 | 0.04582 | 0.029051 | *SYT1* |
| BP | GO:0060448 | dichotomous subdivision of terminal units involved in lung branching | 0.001769 | 0.04582 | 0.029051 | *CTSH* |
| BP | GO:0090716 | adaptive immune memory response | 0.001769 | 0.04582 | 0.029051 | *CD81* |
| BP | GO:1903233 | regulation of calcium ion-dependent exocytosis of neurotransmitter | 0.001769 | 0.04582 | 0.029051 | *SYT1* |
| BP | GO:1903235 | positive regulation of calcium ion-dependent exocytosis of neurotransmitter | 0.001769 | 0.04582 | 0.029051 | *SYT1* |
| BP | GO:1905674 | regulation of adaptive immune memory response | 0.001769 | 0.04582 | 0.029051 | *CD81* |
| BP | GO:1905676 | positive regulation of adaptive immune memory response | 0.001769 | 0.04582 | 0.029051 | *CD81* |
| BP | GO:2000302 | positive regulation of synaptic vesicle exocytosis | 0.001769 | 0.04582 | 0.029051 | *SYT1* |
| BP | GO:2001188 | regulation of T cell activation via T cell receptor contact with antigen bound to MHC molecule on antigen presenting cell | 0.001769 | 0.04582 | 0.029051 | *CD81* |
| BP | GO:2001190 | positive regulation of T cell activation via T cell receptor contact with antigen bound to MHC molecule on antigen presenting cell | 0.001769 | 0.04582 | 0.029051 | *CD81* |
| BP | GO:0001771 | immunological synapse formation | 0.003536 | 0.049497 | 0.031382 | *CD81* |
| BP | GO:0014905 | myoblast fusion involved in skeletal muscle regeneration | 0.003536 | 0.049497 | 0.031382 | *CD81* |
| BP | GO:0035722 | interleukin-12-mediated signaling pathway | 0.003536 | 0.049497 | 0.031382 | *PLCB1* |
| BP | GO:0035739 | CD4-positive, alpha-beta T cell proliferation | 0.003536 | 0.049497 | 0.031382 | *CD81* |
| BP | GO:0036071 | N-glycan fucosylation | 0.003536 | 0.049497 | 0.031382 | *FUT8* |
| BP | GO:0050855 | regulation of B cell receptor signaling pathway | 0.003536 | 0.049497 | 0.031382 | *CD81* |
| BP | GO:0050861 | positive regulation of B cell receptor signaling pathway | 0.003536 | 0.049497 | 0.031382 | *CD81* |
| BP | GO:0060600 | dichotomous subdivision of an epithelial terminal unit | 0.003536 | 0.049497 | 0.031382 | *CTSH* |
| BP | GO:0070498 | interleukin-1-mediated signaling pathway | 0.003536 | 0.049497 | 0.031382 | *PLCB1* |
| BP | GO:0071349 | cellular response to interleukin-12 | 0.003536 | 0.049497 | 0.031382 | *PLCB1* |
| BP | GO:0072674 | multinuclear osteoclast differentiation | 0.003536 | 0.049497 | 0.031382 | *CD81* |
| BP | GO:0072675 | osteoclast fusion | 0.003536 | 0.049497 | 0.031382 | *CD81* |
| BP | GO:1903909 | regulation of receptor clustering | 0.003536 | 0.049497 | 0.031382 | *CD81* |
| BP | GO:1903911 | positive regulation of receptor clustering | 0.003536 | 0.049497 | 0.031382 | *CD81* |
| BP | GO:1904352 | positive regulation of protein catabolic process in the vacuole | 0.003536 | 0.049497 | 0.031382 | *CD81* |
| BP | GO:2000561 | regulation of CD4-positive, alpha-beta T cell proliferation | 0.003536 | 0.049497 | 0.031382 | *CD81* |
| BP | GO:2000563 | positive regulation of CD4-positive, alpha-beta T cell proliferation | 0.003536 | 0.049497 | 0.031382 | *CD81* |
| MF | GO:0008424 | glycoprotein 6-alpha-L-fucosyltransferase activity | 0.001542 | 0.014975 | 0.005647 | *FUT8* |
| MF | GO:0030108 | HLA-A specific activating MHC class I receptor activity | 0.001542 | 0.014975 | 0.005647 | *CTSH* |
| MF | GO:0032393 | MHC class I receptor activity | 0.001542 | 0.014975 | 0.005647 | *CTSH* |
| MF | GO:0032397 | activating MHC class I receptor activity | 0.001542 | 0.014975 | 0.005647 | *CTSH* |
| MF | GO:0046921 | alpha-(1->6)-fucosyltransferase activity | 0.001542 | 0.014975 | 0.005647 | *FUT8* |
| MF | GO:0070324 | thyroid hormone binding | 0.001542 | 0.014975 | 0.005647 | *CTSH* |
| MF | GO:0008289 | lipid binding | 0.001565 | 0.014975 | 0.005647 | *PLCB1/CD81/DYSF* |
| MF | GO:0042289 | MHC class II protein binding | 0.003082 | 0.025812 | 0.009733 | *CD81* |
| MF | GO:0008656 | cysteine-type endopeptidase activator activity involved in apoptotic process | 0.00462 | 0.028141 | 0.010611 | *CTSH* |
| MF | GO:0016505 | peptidase activator activity involved in apoptotic process | 0.00462 | 0.028141 | 0.010611 | *CTSH* |
| MF | GO:1990459 | transferrin receptor binding | 0.00462 | 0.028141 | 0.010611 | *CD81* |
| MF | GO:0004435 | phosphatidylinositol phospholipase C activity | 0.006156 | 0.028625 | 0.010793 | *PLCB1* |
| MF | GO:0140103 | catalytic activity, acting on a glycoprotein | 0.006156 | 0.028625 | 0.010793 | *FUT8* |
| MF | GO:0008047 | enzyme activator activity | 0.00653 | 0.028625 | 0.010793 | *CTSH/PLCB1* |
| MF | GO:0005543 | phospholipid binding | 0.006683 | 0.028625 | 0.010793 | *PLCB1/DYSF* |
| MF | GO:0004629 | phospholipase C activity | 0.00769 | 0.028625 | 0.010793 | *PLCB1* |
| MF | GO:0015485 | cholesterol binding | 0.00769 | 0.028625 | 0.010793 | *CD81* |
| MF | GO:0042287 | MHC protein binding | 0.00769 | 0.028625 | 0.010793 | *CD81* |
| MF | GO:0032934 | sterol binding | 0.012281 | 0.039507 | 0.014897 | *CD81* |
| MF | GO:0001618 | virus receptor activity | 0.013807 | 0.039507 | 0.014897 | *CD81* |
| MF | GO:0008417 | fucosyltransferase activity | 0.013807 | 0.039507 | 0.014897 | *FUT8* |
| MF | GO:0042562 | hormone binding | 0.013807 | 0.039507 | 0.014897 | *CTSH* |
| MF | GO:0140272 | exogenous protein binding | 0.013807 | 0.039507 | 0.014897 | *CD81* |
| MF | GO:0004177 | aminopeptidase activity | 0.015331 | 0.039507 | 0.014897 | *CTSH* |
| MF | GO:0016504 | peptidase activator activity | 0.015331 | 0.039507 | 0.014897 | *CTSH* |
| MF | GO:0140375 | immune receptor activity | 0.015331 | 0.039507 | 0.014897 | *CTSH* |
| MF | GO:0005546 | phosphatidylinositol-4,5-bisphosphate binding | 0.016854 | 0.040328 | 0.015206 | *PLCB1* |
| MF | GO:0043028 | cysteine-type endopeptidase regulator activity involved in apoptotic process | 0.016854 | 0.040328 | 0.015206 | *CTSH* |

^1^BP: biological process; MF: molecular function.

**Table S8** Results of the Scrotal Circumference enrichment analysis from the genes prioritized in the functional annotation study with whole-genome sequence data.

| Ontology ^1^ | ID | Description | p-value | p-adjust | q-value | Gene |
| --- | --- | --- | --- | --- | --- | --- |
| MF | GO:0070330 | aromatase activity | 0.002055 | 0.016179 | 0.003784 | *CYP19A1* |
| MF | GO:0016712 | oxidoreductase activity, acting on paired donors, with incorporation or reduction of molecular oxygen, reduced flavin or flavoprotein as one donor, and incorporation of one atom of oxygen | 0.003595 | 0.016179 | 0.003784 | *CYP19A1* |
| MF | GO:0004497 | monooxygenase activity | 0.014342 | 0.029571 | 0.006917 | *CYP19A1* |
| MF | GO:0020037 | heme binding | 0.01893 | 0.029571 | 0.006917 | *CYP19A1* |
| MF | GO:0046906 | tetrapyrrole binding | 0.021983 | 0.029571 | 0.006917 | *CYP19A1* |
| MF | GO:0005506 | iron ion binding | 0.022999 | 0.029571 | 0.006917 | *CYP19A1* |
| MF | GO:0016705 | oxidoreductase activity, acting on paired donors, with incorporation or reduction of molecular oxygen | 0.022999 | 0.029571 | 0.006917 | *CYP19A1* |

^1^CC: cellular component; MF: molecular function.

**Table S9** Results of the Yearling weight and Post-Weaning Weight gain enrichment analysis from the genes in the functional annotation study with whole-genome sequence data.

| Ontology ^1^ | ID | Description | p-value | p-adjust | q-value | Gene |
| --- | --- | --- | --- | --- | --- | --- |
| BP | GO:0046838 | phosphorylated carbohydrate dephosphorylation | 0.003535 | 0.048963 | 0.025074 | *BPNT2* |
| BP | GO:0046855 | inositol phosphate dephosphorylation | 0.003535 | 0.048963 | 0.025074 | *BPNT2* |
| BP | GO:0071545 | inositol phosphate catabolic process | 0.004417 | 0.048963 | 0.025074 | *BPNT2* |
| BP | GO:0046854 | phosphatidylinositol phosphate biosynthetic process | 0.00706 | 0.048963 | 0.025074 | *BPNT2* |
| BP | GO:0043647 | inositol phosphate metabolic process | 0.00794 | 0.048963 | 0.025074 | *BPNT2* |
| BP | GO:0046174 | polyol catabolic process | 0.00794 | 0.048963 | 0.025074 | *BPNT2* |
| BP | GO:0046164 | alcohol catabolic process | 0.013207 | 0.069808 | 0.035748 | *BPNT2* |
| BP | GO:0006334 | nucleosome assembly | 0.015833 | 0.073226 | 0.037498 | *NAP1L5* |
| BP | GO:1901616 | organic hydroxy compound catabolic process | 0.019325 | 0.073794 | 0.037789 | *BPNT2* |
| BP | GO:0006661 | phosphatidylinositol biosynthetic process | 0.020197 | 0.073794 | 0.037789 | *BPNT2* |
| BP | GO:0034728 | nucleosome organization | 0.021939 | 0.073794 | 0.037789 | *NAP1L5* |
| BP | GO:0019751 | polyol metabolic process | 0.024547 | 0.075686 | 0.038758 | *BPNT2* |
| BP | GO:0065004 | protein-DNA complex assembly | 0.028882 | 0.078618 | 0.04026 | *NAP1L5* |
| BP | GO:0046488 | phosphatidylinositol metabolic process | 0.029747 | 0.078618 | 0.04026 | *BPNT2* |
| BP | GO:0046434 | organophosphate catabolic process | 0.03234 | 0.079766 | 0.040848 | *BPNT2* |
| BP | GO:0046474 | glycerophospholipid biosynthetic process | 0.035789 | 0.079766 | 0.040848 | *BPNT2* |
| BP | GO:0071824 | protein-DNA complex subunit organization | 0.036649 | 0.079766 | 0.040848 | *NAP1L5* |
| BP | GO:0006338 | chromatin remodeling | 0.043514 | 0.089446 | 0.045804 | *NAP1L5* |
| BP | GO:0008654 | phospholipid biosynthetic process | 0.047786 | 0.093057 | 0.047654 | *BPNT2* |
| BP | GO:0045017 | glycerolipid biosynthetic process | 0.052044 | 0.096281 | 0.049305 | *BPNT2* |
| CC | GO:0032588 | trans-Golgi network membrane | 0.007272 | 0.087263 | 0.061237 | *BPNT2* |
| MF | GO:0008441 | 3'(2'),5'-bisphosphate nucleotidase activity | 0.000771 | 0.002699 | 0.001217 | *BPNT2* |
| MF | GO:0097657 | 3',5'-nucleotide bisphosphate phosphatase activity | 0.000771 | 0.002699 | 0.001217 | *BPNT2* |
| MF | GO:0008252 | nucleotidase activity | 0.003082 | 0.007191 | 0.003244 | *BPNT2* |
| MF | GO:0016791 | phosphatase activity | 0.045562 | 0.07568 | 0.034141 | *BPNT2* |
| MF | GO:0042578 | phosphoric ester hydrolase activity | 0.064868 | 0.07568 | 0.034141 | *BPNT2* |

^1^BP: biological process; CC: cellular component; MF: molecular function.
