# Supplementary material for: Genome-wide association study considering genotype-by-environment interaction for productive and reproductive traits using whole-genome sequencing in Nellore cattle
Source: BMC Genomics. 2024 Jun 20;25:623. doi: 10.1186/s12864-024-10520-x (PMC11188527; doi:10.1186/s12864-024-10520-x)
Supplement: Supplementary file 2 — Supplementary Material 2 [file 12864_2024_10520_MOESM2_ESM.docx]

# Additional file 2


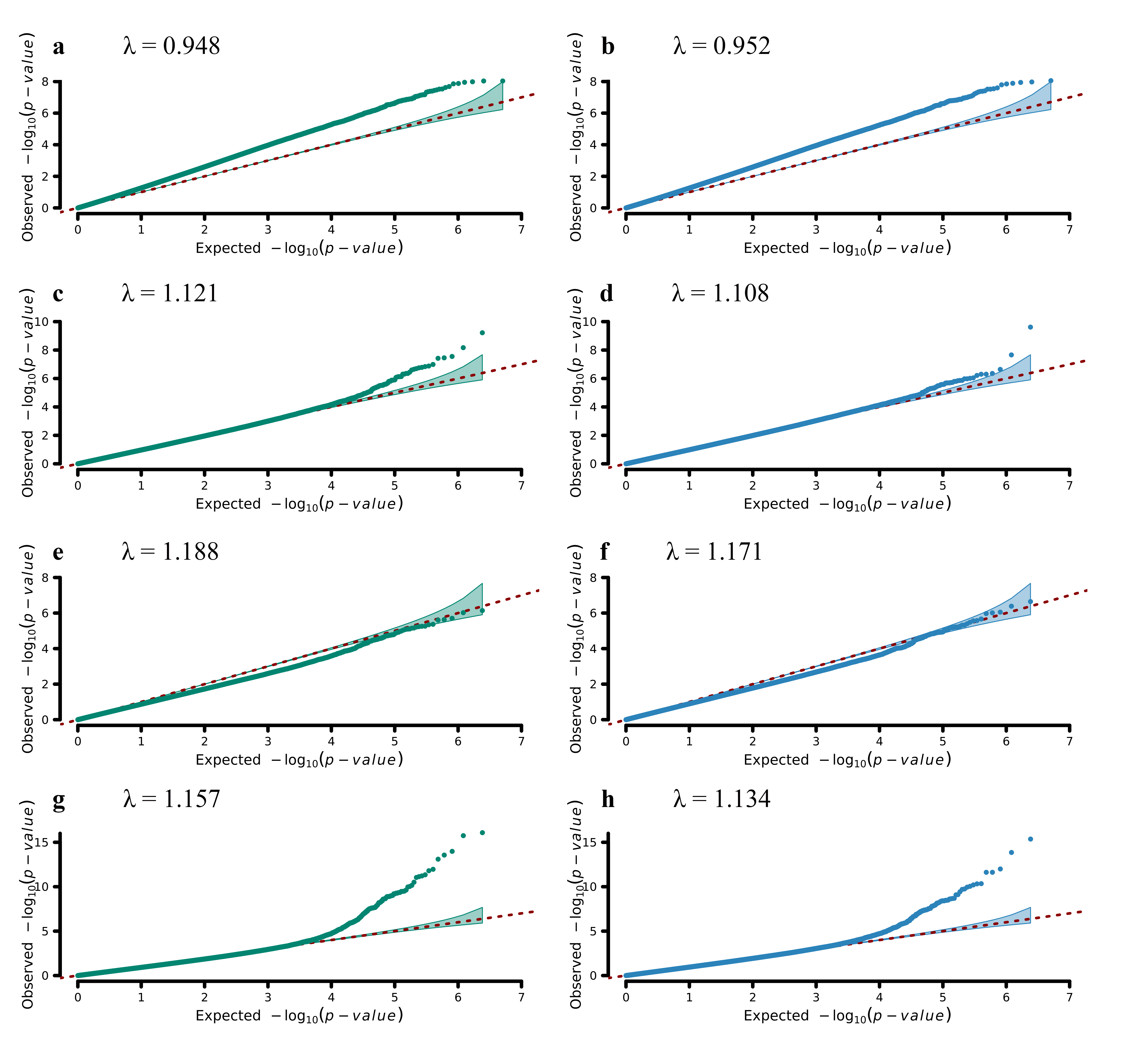


**Figure S1 -** Quantile-quantile (QQ) plot, comparison of primary GWAS ${-log}_{10}\left( p-value \right)$ to those expected for a null distribution. Results for intercept: a - Age at first calving (AFC), c – Scrotal circumference (SC), e - post-weaning weight gain (PWG) and g - yearling weight (YW) and for slope b - Age at first calving (AFC), d – Scrotal circumference (SC), f - post-weaning weight gain (PWG) and g - yearling weight (YW).
